# Phytofabricated silver nanoparticles unlock new potential in tomato plants by combating wilt infection and enhancing plant growth

**DOI:** 10.1038/s41598-025-89724-4

**Published:** 2025-03-27

**Authors:** Hina Ashraf, Tehmina Anjum, Irfan S. Ahmad, Rashid Ahmed, Zill-e-Huma Aftab, Humaira Rizwana

**Affiliations:** 1 Department of Plant Pathology, Faculty of Agricultural-Sciences, University of the Punjab, Lahore, Pakistan; 2 Department of Agricultural and Biological Engineering, University of Illinois at Urbana- Champaign, Illinois Champaign, USA; 3 Holonyak Micro and Nanotechnology Laboratory, University of Illinois at Urbana- Champaign, Champaign, Illinois USA; 4 Department of Biotechnology, Mirpur University of Science and Technology (MUST), Azad Jammu and Kashmir, Pakistan; 5 Department of Botany and Microbiology, College of Science, King Saud University, Riyadh, Kingdom of Saudi Arabia; 6 Department of Analytical Chemistry, The Connecticut Agricultural Experiment Station, New Haven, CT USA

**Keywords:** Silver nanoparticles (AgNPs), *Pongamia pinnata* (PP), Antimycotic activity, Genes, Field trials, Metal analysis, Plant immunity, Nanoparticles, Fungal host response

## Abstract

The environment faces serious threats from climate change, food security challenges, and a growing population. The UN Global Goals emphasize the urgent need for sustainable agriculture to secure food production. We must adopt innovative solutions to bolster agroecological resilience and increase food output with minimal environmental impact. Here, we investigate the antimycotic properties of silver nanoparticles (PP-AgNPs) at various concentrations in controlling Fusarium wilt for tomato crop improvement under laboratory, greenhouse, and field conditions. Various instruments were utilized to characterize the green-synthesized PP-AgNPs. The results indicated a broad UV peak at 428 nm and a spherical morphology with sizes ranging from 1 to 3.5 nm, as confirmed by SEM and TEM. Analyses indicate that the antifungal potency of PP-AgNPs (150 µg/mL) against *Fusarium oxysporum* was found to be 80.9% (Colony diameter: D_A_) and 95.4% (Measured area -M_A_), respectively, in contrast to the control treatment. Notably, the concentration of PP-AgNPs at 100 µg/mL signified the best effect under greenhouse and field trials, reducing disease severity by 34.5% (greenhouse) and 21.8% (Field: average of both years). PP-AgNPs also render other benefits, including improved plant growth parameters, fruit weight, number, and bioactive compounds. After exposure to PP-AgNPs, there was a significant increase in the expression of pathogenicity-related (PR), and defense genes at the molecular level. The physiological and molecular data are in-line induced antioxidative and defense responses after treatment with PP-AgNPs. Furthermore, the Ag content in various parts of tomato plants reveals no adverse effect on plant yield. Current research indicates that PP-AgNPs may be an effective and sustainable product for managing diseases and increasing crop yields in agriculture.

## Introduction

To address global sustainable agriculture challenges, we should explore evidence-based technologies^1^. Nanoscience and nanoparticles can potentially transform the agriculture industry due to their nano-specific characteristics, such as tunable size, morphology, surface chemistry, and great efficiency^2^. Nanotechnology can renovate traditional strategies such as crop fertigation and protection, particularly through the delivery of pesticides and nutrients. Research has shown that nanoparticles can boost crop yield and nutritional quality by combating pathogen infections. The small size of nanoparticles improves their transport within plants, activating defense mechanisms via the systemic acquired resistance (SAR) pathway. For instance, nanoparticulate silver (Ag/Ag–Si) effectively protects tomato plants from foliar pathogens^3^.

So far, literature reported numerous nanoparticles, like Ag, Cu, TiO_2_, SiO_2_, Ag, rGO, and so on. However, silver nanoparticles (AgNPs) are attracting momentous attention from scientific groups and industry because of their immense potential^4^. AgNPs are one of the extensively explored nano-agents because of their broad-spectrum anti-microbial properties and robust inhibition against agriculture pests and pathogens^5^. Approximately 650 various types of microorganisms are being targeted by silver, therefore it can be used in the plant protection division^6^. There are many studies in the literature on the fungistatic activity of AgNPs including *Fusarium graminearum*^7^*, F. verticillioides*^8^*, Alternaria solani*^9^*, Puccinia striiformis*^10^*, Ustilaginoidea virens*^11^ and *Fusarium oxysporum*^12,13^. The antifungal mechanism of silver nanoparticles (AgNPs) primarily involves inhibiting hyphal growth and spore germination, disrupting the cell wall and membrane potential, and generating reactive oxygen species (ROS). This process is mediated by altering stress gene transcript levels, leading to genotoxic effects that damage DNA. Additionally, AgNPs induce protein denaturation by interacting with proteins containing sulfhydryl groups (–SH)^14,15^.

AgNPs are also considered novel growth stimulators in plants as reported to improve biomass, promote germination, intensify the pigment content, and boost growth and fruit quality^16,17^. Additionally, it has also been reported that AgNPs also enhance the activity of certain enzymes such as superoxide dismutase (SOD), peroxidase, and catalase in the roots and shoots of different plants^6^.

Multiple conventional methods exist for the fabrication of nanoparticles (NPs), including physical, chemical, and biological^18,19^. However, the application of physical and chemical processes is limited due to their hazardous consequences^20^. The environmentally friendly synthesis and assembly of nanoparticles, often called ‘green chemistry’ or ‘green pathways,’ typically utilizes various stabilizing and reducing agents derived from microbial sources, plants, and other natural resources. The green synthesis of nanoparticles has gained significant interest recently due to its enhanced stability, cost-effectiveness, and eco-friendly approaches^21^.

The presence of stabilizing agents adsorbed on the surface of AgNPs appears to be the chief factor in modeling bio-activity^22^. Additionally, stabilizers such as biologically active chemicals can enhance the perforation of AgNPs in bio-membranes and facilitate their accretion in cell components^8^. *Pongamia pinnata*, a member of the Fabaceae family indigenous to Asia, is rich in phytonutrients such as flavones and chalcone, available frequently in the leaves and stems of plants^23^. Leaves of *P. pinnata* are harvested for their antimicrobial activities^24^. Earlier investigation revealed the potential activity of antifungal constituents of leaves acts a reducing agent for the synthesis of nanoparticles^25^.

*Fusarium oxyporum* f.sp. *lycopersici* (FOL)*,* the causal agent of the tomato wilt, is a destructive ascomycete fungal pathogen, documented in over 30 countries, instigated infection both in greenhouse and field environments^26^. FOL significantly contributes to the reduction in tomato crop production, causing a global fall of 10–50%. In particular, warmer locations like Pakistan have yield losses ranging from 10 to 90%^27^. Managing soil-borne pathogens has long been an uphill battle within the agricultural system. While several solutions have been developed to address the infection caused by *F. oxysporum*, these approaches are not devoid of limits.

This investigation aimed to highlight the potential of green-synthesized PP-AgNPs in suppressing wilt infection and enhancing growth characteristics in tomato plants. This study was conducted under In-vitro*,* greenhouse, and field conditions. The antimicrobial activity was assessed by measuring inhibition zones and observing morphological changes in fungal hyphae. Plant growth parameters, disease attributes, and fruit development were also evaluated. To better understand the effects of PP-AgNPs, we analyzed the physiological and molecular responses of tomato plants by examining oxidative, antioxidative, and defense enzymes. Furthermore, field studies were carried out over two consecutive years, using the most effective concentration of PP-AgNPs. Silver content was also measured in different parts of the tomato plants (roots, shoots, and fruits) to ensure compliance with food safety recommendations.

## Materials and methods

### Reagents and materials

All reagents were of analytical grade, obtained from Sigma-Aldrich, and used without further purification. All solutions of silver nitrate (AgNO_3_, 99.0%), sodium hydroxide (NaOH), and Potato dextrose agar (PDA) were prepared with deionized water (resistivity $\nless$ 18.2 MΩ cm^−1^).

During the springtime, young and fresh *Pongamia pinnata* L. (PP) leaves were taken from the experimental station of the Department of Plant Pathology, University of Punjab (DPP-PU), Lahore. Solar-dried leaves (20 g) were boiled (100 mL deionized water) for 30 min at 90 °C in a digital thermostat water bath. After cooling, the plant extract underwent filtration using Whatman filter paper at ambient temperature. The sample was stored under refrigeration conditions and utilized within 2 weeks for further empirical studies. A pathogenic strain of *Fusarium oxysporum* f. sp. *lycopersici* (IAGS-1322), originally isolated from the roots of infected tomato plants, was procured from FCBP (Fungal culture Bank of Pakistan), stored (4 °C), and cultured in glass tubes for subsequent experiments.

### Synthesis and optimization of Pongamia pinnata (*PP*) capped silver nanoparticles (PP-AgNPs)

The typical reaction for green synthesized silver nanoparticles (PP-AgNPs) was carried out in a 100 mL flask, involving the stepwise addition of 5 mL of *P. pinnata* leaf extract to 45 mL of 2 mM silver nitrate (AgNO_3_). The pH of the reaction mixture was adjusted at 10 through the dropwise addition of sodium hydroxide (1 M). Subsequently, the reaction was subjected to microwave irradiation for a pulse of 90 s for rapid synthesis. Eventually, a blackish-brown color was formed, which indicated the formation of PP-AgNPs. The reaction conditions for the synthesis of PP-AgNPs were optimized by maintaining pH (6, 8, 10), silver nitrate concentration (0.5, 1, 2, 3), amount of PP-leaf extract (1, 3, 5, 7), microwave irradiation (5, 10, 15, 20, 30, 45, 60, 75, 90, 105, 120). Dark conditions under ambient temperature were used to perform all reactions. The details of techniques employed to characterize the PP-AgNP were discussed in experiment S1*.

### Antimycotic effect of PP-AgNPs on mycelial growth

The agar dilution technique was employed to investigate the antimycotic activity of green synthesized PP-AgNPs against *Fusarium oxysporum*. An aliquot of stock solution was added to the media (PDA) to prepare final concentrations of 50, 100, 125, and 150 µg of PP-AgNPs per mL. Aseptically, 4 mm diameter PDA plugs that the fungus had colonized were excised from the outer region of 7-days-old *F. oxysporum* culture plates and then introduced into the central region of individual plates. Media plates with distilled water only serve as a positive control, whereas those with fungicide (0.1% of Nativo-WG 75) are used as a negative control. Each treatment was replicated thrice. The plates with fungal discs were incubated under intermittent dark and light conditions at 25 °C until the control plates were colonized to the margins. For measuring the fungal colony, plate images were captured and analyzed using ImageJ 1.53 k to determine the measured area of the fungal colony (M_A_). According to Hendrick’s^28^ method, the average diameter (DA) was changed to the area of a circle using the following formula.

$$D_{A} = \pi r^{2} = \pi \left( {Average\;D/2} \right)^{2}$$

The percentage inhibition for PP-AgNPs hyphal growth of *F. oxysporum* was calculated using the control (PDA only) and PDA amended with various concentrations of nanoparticles and fungicide by using the formula:

$$Percentage\; Inhibition \left( \% \right) = \frac{Average \;Control \;Area - Average \;Treated \;Area }{{Average\; Control\; Area}}$$

Experiment S2* presents a detailed methodology for evaluating the effect of PP-AgNPs on ultrastructural, plasma membrane, cell-wall integrity, and ROS generation in *F. oxysporum.*

### In-planta activity of PP-AgNPs on disease activity and plant growth parameters

*In-planta* studies were performed to assess the antimycotic effect of PP-AgNPs on *F. oxysporum* causal agent of Tomato wilt. The wilt-susceptible variety of tomatoes, i.e., Early Boy, was used as a model plant for greenhouse trials. All details of an experimental setup for greenhouse studies were reported in our earlier work^29^. The harvest was carried out after 45 days of being incubated in a greenhouse. Five random plants were chosen from each treatment, and information about growth characteristics such as plant height, length of roots and shoots, and fresh and dry biomass were recorded. Dry biomass (g) was calculated by maintaining the seedlings in a vacuum oven at 60 °C for 3 days. In contrast, fresh biomass (g) was calculated by removing excess moisture from the seedlings along with their roots. The following formula was used to assess the disease incidence following the application of various PP-AgNP treatments on tomato plants^30^.

$$Disease \;Incidence \left( \% \right) = Infected \;plants/Total \;plants \times 100$$

The severity of the disease was evaluated using a modified 0–6 rating scale^31^. To calculate the percentage disease severity (PDS), the following equation was applied:

$$Disease\; Severity \left( \% \right) = \sum \left\{ {\left( {\eta \times {\mathcal{V}}} \right)/\left( {6 \times {\text{N}}} \right)} \right\} \times 100$$

To determine the response of different tomato varieties against fusarium wilt, a scale developed by Popoola^32^ was used. The scale measures the numerical grade of infected plants (V) and the total number of examined plants in each treatment (N). The highest grade of infection category is 6.

Using this disease rating scale, the following values of PDS (percentage disease severity) indicate different levels of resistance: 0% = immune; 1–5% = resistant; 6–10% = moderately resistant; 11–25% = moderately susceptible; 26–70% = susceptible and > 75% = highly susceptible.

The fruits were collected on the 145th day after transplantation, ensuring they were uniform and undamaged. The average weight and number of fruits per plant were recorded.

### Evaluating bioactive compounds in fruits

Four uniformly sized tomatoes with a light to dark red color were collected at full maturity to ensure consistency and avoid physical deformities. The total protein content in the fruit was estimated using the Lowry method^33^, with bovine serum albumin serving as the standard. To quantify flavonoids, the methodology of Arvouet-Grand^34^ was followed involved the use of aluminum trichloride at 415 nm. Protocol of Fish^35^ was used to measure the lycopene content in the fruit by measuring absorbance at 503 nm. The amount of vitamin C in the fruit was calculated using 2,6-dichlorophenol and 2% hydrochloric acid^36^. The protocols for quantification of stress enzymes in the root and shoot of tomato plants exposed to various concentrations of PP-AgNPs are discussed in Experiment S3*. Gene-expression studies to evaluate the transcript level of pathogenicity (PR2 and PR5) and defense-related genes (PPO, PAL, POD, CAT, SOD) after exposure to 100 µg/mL of PP-AgNPs are elucidated in Experiment S4*.

### Field efficacy of the most effective treatment of PP-AgNPs

Field studies were conducted twice during the tomato growing season, which took place from October through March 2021–2022, at the experimental station of the Department of Plant Pathology, University of the Punjab, Lahore, Pakistan. The field experiment was carried out using a single row-plot design. Tomato seeds of the “Early Boy” variety were planted in soil that had been sterilized. A 100 g/row fungal inoculum of *F. oxysporum* was grown on sorghum grains and added to the sterilized soil to ensure pathogen colonization. The inoculum was then left for a week. The seedlings’ roots were treated for 2 h with the highest concentration of PP-AgNPs, sterile water (positive control), and fungicide (negative control), respectively. The tunnel was approximately three to four meters high. The tomato plants were transplanted and received two foliar spray treatments with the same concentration of PP-AgNPs, scheduled 10 days apart after 2 weeks. The plants were fertilized every 4 weeks with NPK (20:20:20) soluble fertilizer at a 1 g/L rate. Pots were regularly hand-weeded and irrigated, and the plants were checked frequently for signs of disease. After a month of seedlings being transplanted in the field, data were recorded regarding the disease index and growth parameters, such as plant height and total fresh and dry mass. Each plant was harvested at the end of 3 months (90 days), and the total number of fruits and tomato yields were noticed. The processes to estimate silver content in various parts of tomato plants after treatment with PP-AgNPs were explicated in Experiment S5*.

### Statistical analysis

Statistical analysis was carried out using Statistic 8.1 and GraphPad Prism (8.4.3) software from California, USA. Each experiment was performed in triplicate with independent replication, and the results were presented as mean ± standard error (SE) graphs. Group differences were determined using the analysis of variance test (one-way-ANOVA). Significant differences between treatments for plant growth, fruit, and disease attributes were indicated by different superscripted letters, as determined by LSD. Tukey’s multiple comparison tests by GraphPad Prism evaluated the means of other parameters, such as antifungal and enzymatic activity. The Fisher’s Test (*p* < 0.05) was used to determine statistical significance (*P < 0.05, **P < 0.01, *** *P* < 0.05, ns; non-significant difference). The particle size distribution was obtained by histogram using Origin 2018 software, while Image-J (version 1.53f.) was used to determine particle size.

## Results and discussion

### Visual and UV–VIS spectroscopic analysis at varied optimization parameters

**Fig. 1:**
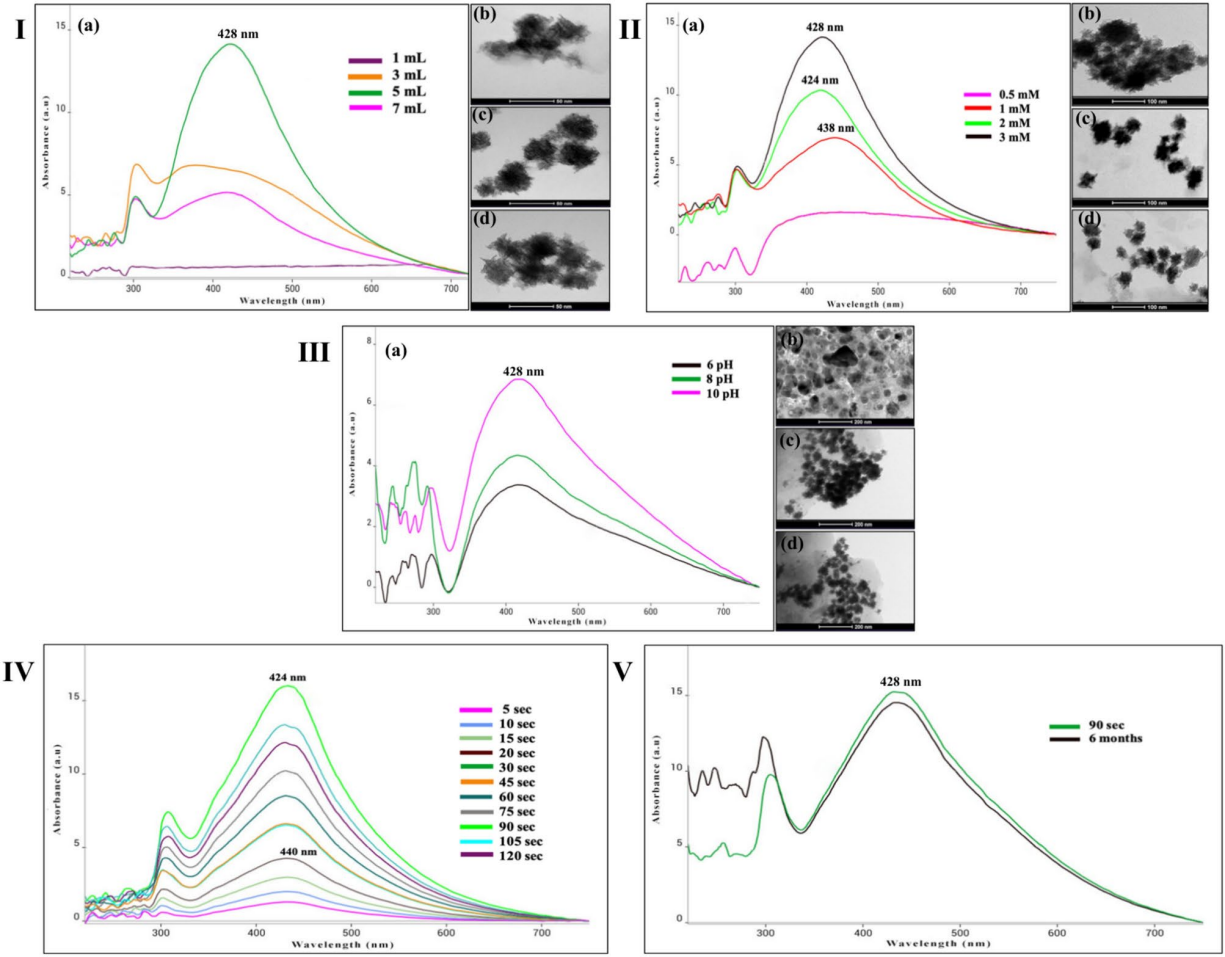
Optimization of reaction conditions for synthesizing microwave-assisted PP-AgNPs imaged by UV–visible spectrophotometer and TEM. **I–III:** represents UV–visible absorption spectra (**a**) and TEM images (**b**–**d**) of PP-AgNPs documented as a function of the amount of PP-leaf extract (1–7 mL), silver nitrate concentrations (0.5-3 mM) and pH (6–10), **IV–V:** microwave-irradiations (5–120 s) and stability (90 s and 6 months).

The bio-reduction of Ag^+^ ions to AgNPs after exposure to *Pongamia pinnata* (PP) extract was initially evidenced from the color variation of the reaction mixture from neutral to yellowish-brown after microwave-irradiation of 90 s due to of excitation of vibration bands related to surface plasmon resonance (SPR) in the visible region of AgNPs^37^. The synthesis of PP-AgNPs was substantiated by a broad absorption peak (*λ*_max_) ranging from 424 to 440 nm, indicating the successful formation of silver nanoparticles in the desirable range. Interestingly, some earlier studies also reported the absorption of microwave-assisted AgNPs between 405 and 440 nm, respectively^38,39^. Figure 1I–V shows the optimization of reaction conditions for microwave-assisted PP-AgNPs imaged with UV–VIS spectroscopy and HR-TEM. The UV–visible spectra shown in Fig. 1I-a depicted the effect of varying volumes of PP extract on the biosynthesis of PP-AgNPs. It is evident from the spectra that the amount of PP at 1 mL signifies no wavelength with decreased particle dimension. An increase in extract volume (3–7 mL) indicated a broad SPR band owing to the agglomerative solution of nanoparticles^40^. Maximum peak intensity for PP-AgNPs was seen at 5 mL of PP extract. The TEM images are in good agreement with UV–visible absorption spectra (Fig. 1I-b–d). From these outcomes, it could be concluded that the optimum amount of PP extract for the synthesis of AgNPs is 5 mL. Similar findings were also reported^41^ with leaf extract of *Azadirachta indica.*

Figure 1II-a presents the effect of different concentrations of silver nitrate (0.5–3 mM) on the synthesis of PP-silver nanoparticles. The absorption spectra indicate no characteristic peak of AgNPs at a lower concentration of AgNO_3_ (0.5 mM); a broad band at 438 nm was perceived, and the spectra endured a blue shift to 424 nm with a high concentration of 2 mM. Further, the rise in the AgNO_3_ concentration triggered a shift to a longer wavelength (428 nm) due to an increase in the diameter of the particle, attributed to aggregation of nanoparticles^42^ that was further proved by TEM images (Fig. 1II-b–d). Almost spherical-shaped monodispersed particles were observed at higher concentrations^43^.

The pH of the reaction solution was maintained by adding PP extract (5 mL) to 2 mM silver nitrate (45 mL) was observed at 10. The absorbance spectra and parallel TEM images (Fig. 1-IIIa–d) exhibited more homogenously spherical AgNPs at higher pH which are to previous findings^44^. Moreover, it was observed that the high pH of the reaction mixture accelerates the reduction rate of AgNO_3,_ and oxidation of metabolites as compared to lower pH, rapidly turned the solution to a colloidal brown color. Thus, the alkaline pH of the reaction mixture is more conducive to the synthesis of AgNPs^45^. Furthermore, at lower pH polydisperse (Fig. 1III-a) AgNPs were formed. Reaction time immensely escalates the absorbance intensity of the reaction mixture. The biosynthesis of PP-AgNPs started within 5 s of microwave irradiation which showed an SPR band at 440 nm. The band intensity escalates with sustained reaction along with a blue shift to 424 nm attributed to the creation of diminutive, spherical-shaped nanoparticles. The microwave synthesis was completed within 90 s^46^ with no further increase in SPR band intensity (Fig. 1IV). Also, even after 6 months, no substantial color and UV spectrum variation was detected, revealing the stabilization of PP-AgNPs in the colloidal suspension (Fig. 1V).

### Characterization of PP-AgNPs by using various techniques

#### Functional Characterization of PP-AgNPs

**Fig. 2:**
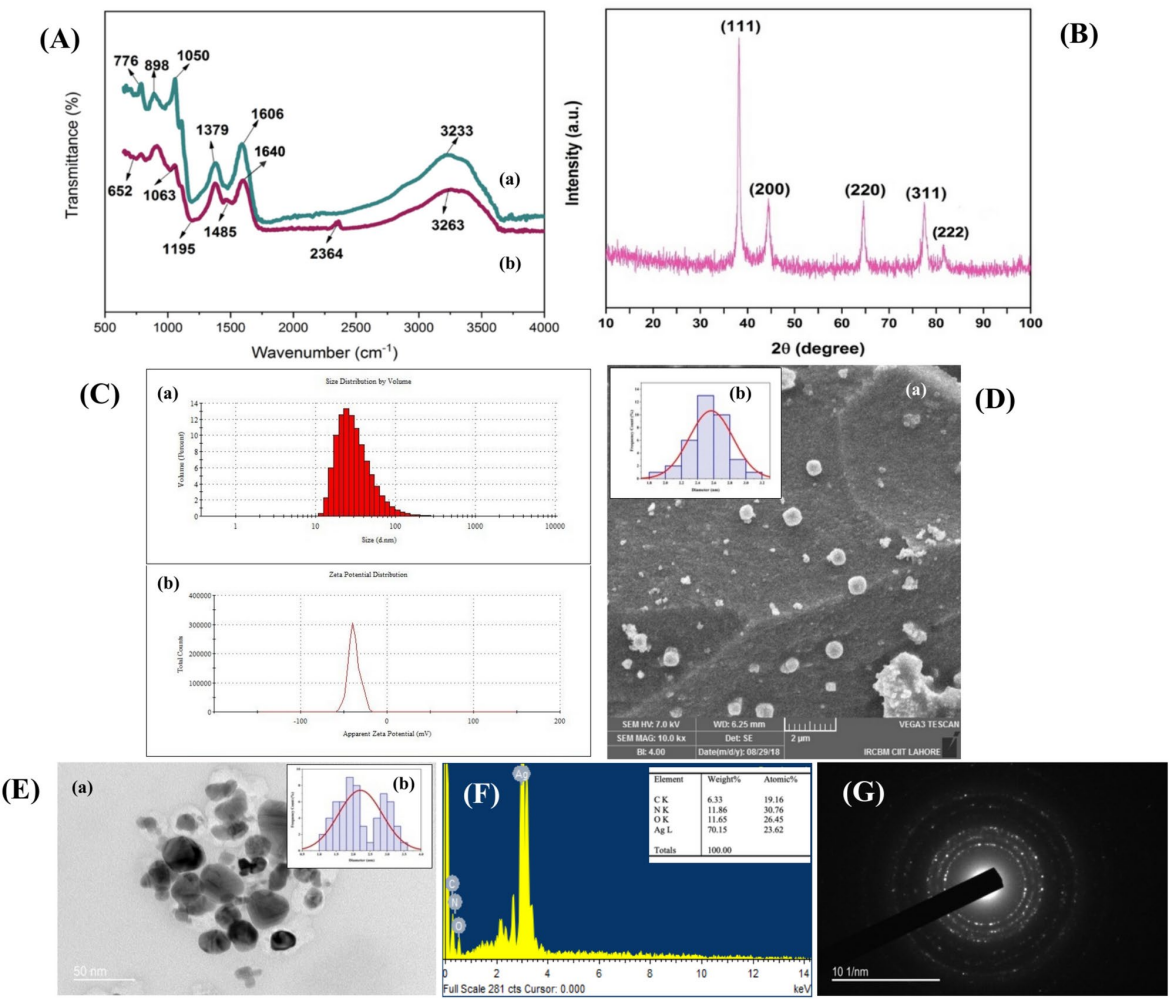
Characterization analysis of microwave-assisted PP-AgNPs by various techniques (**A**) represents FTIR spectra of PP leaf extract (**a**) and PP-AgNPs (**b**); (**B**) XRD pattern of PP-AgNPs; (**C**) Size distribution intensity and zeta potential of PP-AgNPs (**a**&**b**); (**D**) SEM image (**a**) along with size distribution histogram (**b**) of PP-AgNPs (scale bar:2 µm); (**E**) TEM image (**a**) along with size distribution histogram (**b**) of PP-AgNPs (scale bar:500 nm); (**F**) EDX spectrum of PP-AgNPs; (**G**) SAED Pattern of PP-AgNPs.

FTIR spectra of *P. pinnata* (PP) leaf extract and synthesized silver nanoparticles are displayed in Fig. 2A-a,b. Silver ions are reduced to silver nanoparticles by phytochemicals available in PP leaf extracts, which also serve as stabilizing agents to prevent nanoparticle agglomeration^47^. The FTIR spectra of the PP leaf extract are displayed in Fig. 2A-a. The broad peak at approximately 3233 cm^−1^ is associated with the stretching vibrations of the hydroxyl (–OH) group found in aliphatic and phenolic structures. The C=C and C–N stretching in the aromatic ring were detected at 1606 cm^−1^ and 1379 cm^−1^, respectively. The peak at 1050 cm^−1^ corresponds to the stretching vibrations of the ester group bond, while the peak at 898 cm^−1^ is attributed to the C–O groups linked to β glycosidic linkages. The presence of COH stretching vibrations was detected at approximately 776 cm^−1^. Thus, these functional groups of the phytochemical constituents (tunicatachlacone, galactoside, pongaflavanol and pongamol, glybanchalcone) available in leaf extract of *P. pinnata* are primarily liable for synthesis and steadiness of AgNPs^48^. All FTIR spectral analyses are in line with earlier studies^49,50^. Likewise, the presence of phenolic OH may be the reason for the broad peaks in the PP-AgNPs’ FTIR spectrum (Fig. 2A-b) at approximately 3263 cm^−1^, which shows the stretching of the O–H group. The carbon dioxide O=C=O strong stretching that was associated with the peak intensity observed at 2364 cm^−1^ in AgNPs was not present in the PP extract. Strong C–O stretching of primary alcohol was assigned to the peak at 1063 cm^−1^, while the absorption band at approximately 1195 cm^−1^ indicated stretching vibrations of –O–CH_3_. The strong stretching of the alkyl-halide groups’ C–Br was correlated with the minor peak that emerged at approximately 652 cm^−1^. On the surface of PP-AgNPs, the two main peaks at 3263 cm^–1^ and 1640 cm^–1^ revealed the presence of flavonoids, alkaloids, terpenoids, and phenolic compounds, among other phytochemicals. These compounds reduce Ag^+^ to Ag^0^ and form transitional complexes with functional groups^51^.

#### Structural characterization of PP-AgNPs

The nano-crystalline nature escorted by distinct structural peaks of silver nanoparticles was evaluated by XRD pattern^52^. Figure 2B displays the XRD diffractogram of microwave-assisted PP-AgNPs. The main characteristics of silver (Ag) can be observed from the diffraction peaks at (2θ) 38.22, 44.43, 64.58, 77.43, and 81.32, which correspond to the crystallographic planes (111), (200), (220), (311), and (222). The diffraction peaks of PP-AgNPs indicate that they have a face-centered cubic crystal structure similar to that of JCPDS no. 04-078. This suggests that the silver nanoparticles synthesized are of the highest quality. Similar XRD peaks for AgNPs were observed using the *Hagenia abyssinica* plant extract^47^. The peak at 2θ = 38.22 (111) had the highest intensity, indicating a mean particle size of 16 nm determined using Scherrer’s formula for PP-AgNPs. The detection of no extra peaks in XRD spectra signifies the purity of PP-Ag nanoparticles^53^.

#### Surface properties of PP-AgNPs

Dynamic Light Scattering (DLS) and Zeta potential were used to get information regarding surface charge, stability, and size distribution of nanoparticles in colloidal suspension. The average zeta sizer analysis of microwave-assisted PP-AgNPs indicated a size distribution value of 56.9 nm with 0.304 PDI value (Fig. 2C-a). In contrast, the average zeta potential value was equal to – 38.0 mV (Fig. 2C-b). The higher negative zeta-potential value is explained by the presence of naturally occurring stabilizing agents in plant extracts. Strong electrostatic repulsive forces among the particles, which prevent agglomeration and flocculation in liquid media, also denote the long-term stability and quality of NPs^54^. Since DLS measured the diameter of the nanoparticles, which also included water and biomolecules coating the surface of the NPs, hence the size determined by DLS is greater than that determined by XRD, SEM, and TEM^55^. Correspondingly^56^, microwave-irradiated biosynthesized AgNPs expedited quick capping rate and homogenous nucleation that considerably affect nanoparticle size, structure, and dispersity^56^.

#### Morphological characterization of PP-AgNPs

SEM and TEM micrographs investigated the surface morphology and size of microwave-assisted PP-AgNPs, as shown in Fig. 2D,E-a. The images for PP-AgNPs revealed spherical to globular polydisperse, non-agglomerated particles with uniform size distribution^57,58^. The mean particle size obtained by SEM and TEM histograms was found to be 2.50 ± 0.03 nm and 1.89 ± 0.09 nm, respectively (Fig. 2D,E-b). Small particle sizes of PP-AgNPs elicit the process of nucleation, leading to reduction throughout the process^41^. Elemental analysis of PP-AgNPs was shown by the EDX spectrum in Fig. 2F. The strong peak at 3.0 keV indicated the absorption of metallic silver nanocrystals attributed to the SPR^59^. The biosynthesized PP-AgNPs were synthesized with less impurity, indicating the percentage of elemental silver from 70.15% and 23.6%, respectively. Elements such as C, O, and N were also detected in the EDX spectrum, indicating the presence of organic compounds in the reaction mixture of PP-AgNPs that function as capping agents to prevent agglomeration^60^. The corresponding SAED pattern of the PP-AgNPs is shown in Fig. 2G, the faced-centered cubic (fcc) structure of silver indexed by a circular diffraction pattern. Ag (111) and (220) were found to be at a distance of 0.23 nm within the silver particle matrix^37^. The high crystalline nature of microwave-assisted PP-AgNPs was confirmed by the bright circular rings that aligned with the (111), (200), (220), (311), and (222) lattice fringes of fcc Ag^61^.

### In-vitro antimycotic activity of PP-AgNPs on mycelial development of *F. oxysporum*

**Fig. 3:**
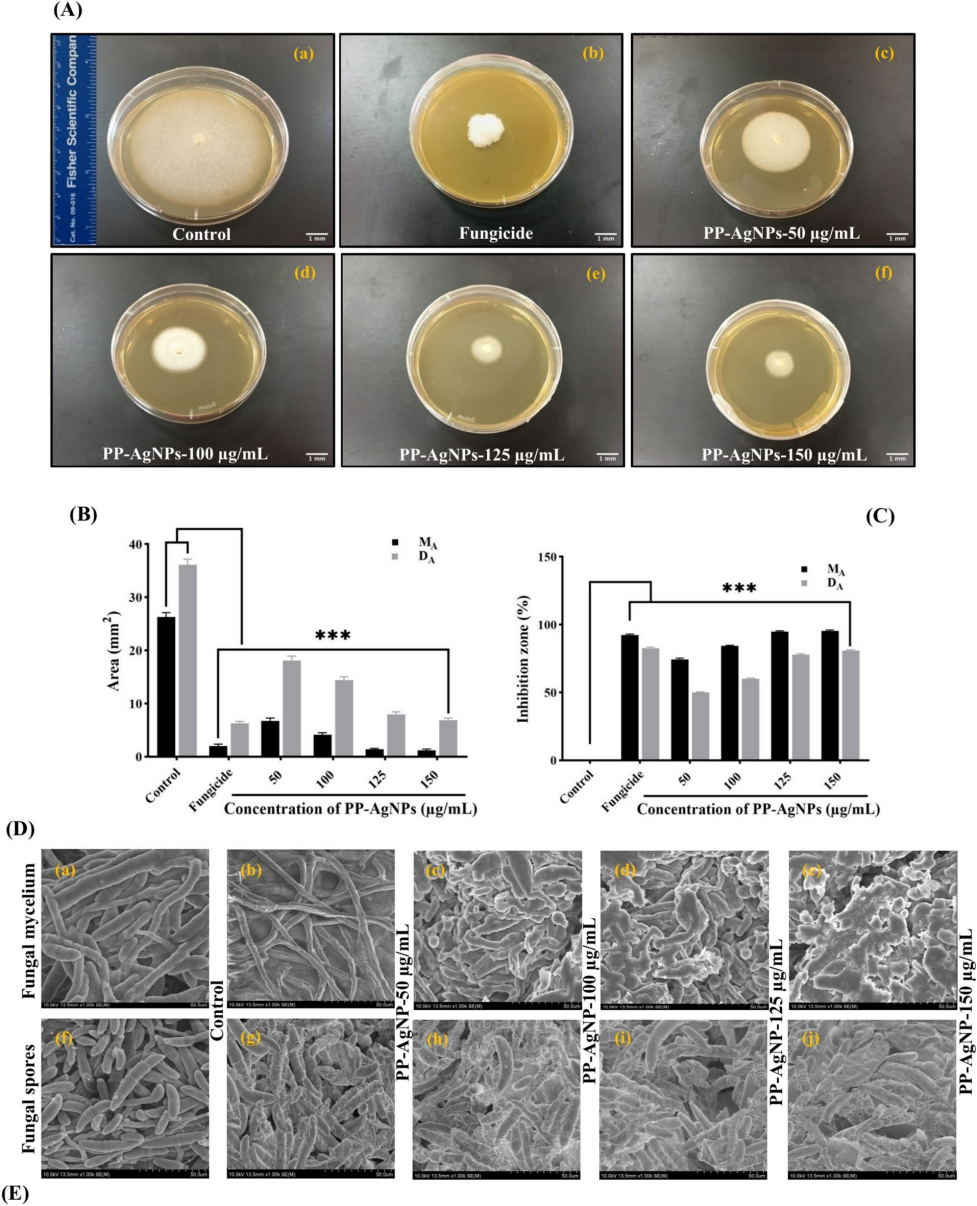
Antimycotic effect of PP-AgNPs on *F. oxysporum* at various concentrations (50–150 μg/mL) in parallel to the control and fungicide treatment after 7 days of post-incubation at 28 °C. (**A**) Shows antifungal effect of PP-AgNPs on mycelial radial growth on PDA media plates; (**B**) Represents area of the fungal growth measured (M_A_-dark bar) and average diameter (D_A_-light bar); (**C**) Illustrates percentage inhibition zone graph; (**D,E**) SEM monographs of *F. oxysporum* (**a**–**e**) mycelium, (**f**–**j**) spores treated with various concentrations of AgPP-NPs, while sterile water was used for control treatment. Red arrows indicate the morphological deformations in fungal mycelia and spores after treatment with nanoparticles. Scale bar: 50 μm. Data represents a mean SD (n = 3) of three replicates indicating significant difference (*** *p* < 0.001; ns: non-significant) as compared to control by two-way-ANOVA (*P* < 0.05) and Dunnett’s-multiple comparing tests.

Owing to their higher antimycotic properties, various nanoparticle types are considered good substitutes for managing economically important phytopathogenic fungi^62^. The effects of PP-AgNPs at different concentrations (50–150 µg/mL) on *F. oxysporum* mycelial growth 7 days after inoculation, in parallel with fungicide treatment and control, are shown in Fig. 3A. It is manifest from the results that radial growth of the fungal colony significantly reduced in a dose-dependent manner of PP-AgNPs Fig. 3A-c–f. By comparing assessing methods for fungal growth in control media and media amended with various concentrations of PP-AgNPs, the total area and inhibition zone were highly significant, with *p*-values of *p* < 0.05. Analysis of fungal area (Fig. 3B) was sufficient to extricate suggestive differences in growth inhibition by nanoparticles for *F. oxysporum* by calculating colony diameter (D_A_) and measured area (M_A_). At the highest concentration (150 µg/mL), a sharp decline in M_A_ (1.22 ± 0.22 mm^2^) and D_A_ (6.87 ± 0.41 mm^2^) was noticed. However, M_A_ and D_A_ were shown to be more subtle at lower concentrations, i.e., 6.72 ± 0.55 and 18.1 ± 0.85 mm2, respectively, in contrast to the control treatment. Correspondingly, fungicide treatment revealed 1.99 ± 0.41 and 6.28 ± 0.37 mm^2^ of M_A_ and D_A,_ indicating a significantly lower fungal area after treatment with PP-AgNPs. The percentage inhibition zones were interestingly minimized after treatment with PP-AgNPs after 7 days of post-inoculation, correspondingly to the above results for M_A_ and D_A_, the highest treatment, 150 µg/mL, reduced the growth of *F. oxysporum,* by 95.4 ± 0.65% (M_A_) and 80.9 ± 0.53% (D_A_). However, a relative decrease (M_A_-74.4 ± 0.91% and D_A_-49.4 ± 0.53%) was noticed for the lowest concentration, i.e., 50 µg/mL, in appraisal to the control treatment. Whereas fungicide Nativo (M_A_-92.4 ± 0.7% and D_A_-82.6 ± 0.85%) witnessed a strong inhibitory effect on the mycelial growth of test fungi, comparable to the highest treatments. A recent review article has reported that silver nanoparticles can instigate extensive impairment of fungal hyphae by penetrating the cell membrane and disrupting cell integrity^63^. Furthermore, it is believed that the small size of AgNPs was responsible for disrupting the functioning of the cell membrane and the synthesis of ergosterol, which altered the osmotic balance and permeability of the membrane, ultimately leading to cell death^64^. It was reported that green synthesized AgNPs exhibited significant inhibition against *F. oxysporum*, indicating that the plant extract contains essential secondary constituents that enhance the antimicrobial activity of silver nanoparticles^65^.

### PP-AgNPs stimulate morphological alterations in *F. oxysporum*

Emancipation of silver ions after interaction of the fungal cell surface with AgNPs disrupted cell replication and intracellular homeostasis, leading to cell death^66^. SEM analysis of the microstructural changes in *F. oxysporum* revealed significant changes in the morphology of the spores and mycelia upon treatment with PP-AgNPs at different concentrations (Fig. 3D,E).

The SEM images (Fig. 3D-a–e) for untreated mycelia (control-sterile water) showed cylindrical morphology with a smooth exterior surface and uniform thickness. In contrast, treated mycelia appeared as deformed, irregular, wrinkled, and stacked with ruptured walls. However, mycelial impairment was intensified at higher concentrations (Fig. 3D-c–e), indicating collapsed structures, rifts, or blebs with the presence of minute granules such as polys. Likewise, the mycelia spore morphology of *F. oxysporum* was also altered with increased concentrations of PP-AgNPs (Fig. 3E-f–j). Ultra-structural inspection found that fungal spores treated with sterile water (control) retained a naturally intact and relatively smooth surface with curved to sickle ends of macroconidia and perpetual cytoarchitecture (Fig. 3E-f)^67^. However, as depicted in Fig. 3E-g–j, after exposure to different concentrations of PP-AgNPs, the spores became irregular, withered, and amassed to form a bumpy structure. Our SEM results indicated that AgNPs were accumulated on the surface of fungal cells, which specifies that this accretion may instigate inhibition and lysis of spores^8,68^. Additionally, dispersed silver NPs have been suggested as a key factor in antifungal activity. Some previous studies have reported a delay in mycelial development after treatment with biosynthesized silver nanoparticles^69,70^. In this case, the disruption of fungal hyphae may be linked to changes in cell wall composition due to the inhibition of essential enzymes involved in cell wall synthesis^71,72^.

### Fluorescence microscopy of *F. oxysporum* after treatment with PP-AgNPs

The fungicidal mechanism of PP-AgNPs against *F. oxysporum* was systematically assessed through the examination of reactive oxygen species (ROS) accumulation, membrane permeability, and the synthesis of chitin or glucan after the absorption or retention of specific dyes.

Figure S1(A–O) illustrates fluorescence microphotographs of the untreated (control) and treated (50–150 µg/mL of PP-AgNPs) mycelium of *F. oxysporum.* Multiple researchers have reported using DCFH-DA dye to detect intracellular ROS generation in fungal mycelium induced after treatment with AgNPs^73^. Mycelial samples treated with PP-AgNPs showed increased fluorescence expression (Figure S1B–E). The control samples displayed either very weak fluorescence or none at all, while in contrast, the samples treated with PP-AgNP displayed significant fluorescence (Figure S1A). Additionally, the fluorescence intensity increased at higher PP-AgNP concentrations (Figure S1D&E). It is believed that AgNPs have a potent antifungal effect since they can increase ROS levels, causing damage to cell components and ultimately inhibiting fungal growth^74^. A red fluorescence-emitting PI dye was used to measure the membrane integrity of *F. oxysporum*, indicating dead cells with damaged membranes^75^.

As shown in Figure S1H–J, the fluorescence intensity was substantially higher in the PP-AgNPs treated samples than in the control (Figure S1G). Disruption of membrane permeability and leakage of ions and proteins interceded the morphological alternations in the fungal membrane^76,77^. Our findings align with earlier work^8^ suggesting that treatment with AgNPs increases red fluorescence in *F. verticilliodes* and stimulates cell membrane injury or permeability. Figure S1K–O shows the calcofluor white staining of glucan and chitin in *F. oxysporum* cell walls after structural distractions induced by PP-AgNPs. The calcofluor white staining of chitin and glucan in *F. oxysporum* cell walls sticking to structural distractions induced by PP-AgNPs is displayed in Figure S1K–O. Dark blue fluorescence control cells emit^75^ the intact mycelium with a septation (Figure S1K). In contrast, the samples treated with PP-AgNPs exhibit reduced levels of chitin and glucan within the fungal cell wall (Figure S1L–O).

Our results indicate that PP-AgNPs degraded the fungal cell wall in a dose-dependent manner, consistent with previous findings^29^ using copper oxide nanoparticles.

### In-planta activity of PP-AgNPs on disease attributes and growth parameters of tomato

The novel approach of utilizing AgNPs as an alternative to commercial fungicides has proved to be more efficient. Effectively, they may be implemented as part of plant disease management strategies^78^. Recently, the demand for antifungal agents has increased rapidly due to the development of resistant mutants in response to irregular application of agrochemicals, which alters the fungal generations^79,80^.

**Fig. 4:**
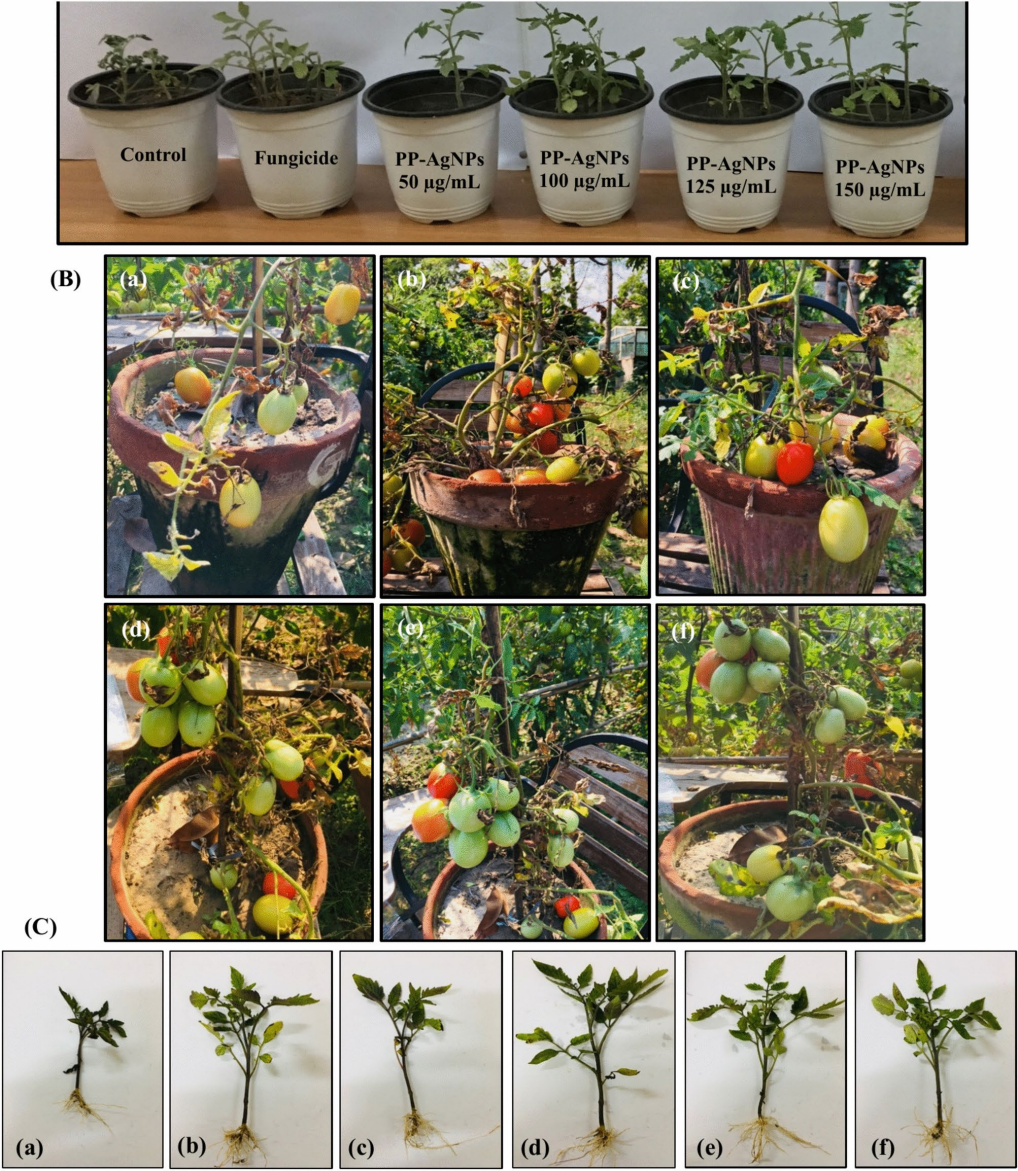
Effect of different concentrations of PP-AgNPs (50–150 µg/mL) along with control and fungicide treatments on tomato growth parameters under greenhouse conditions by inhibiting the wilt infection of *Fusarium oxysporum*, (**A**) represents the growth of plant seedlings in small plastic pots, (**B**(**a–f**)) showing growth of tomato fruits after treatment with PP-AgNPs at 145^th^ day to calculate fruit weight and number, (**C**(**a–f**)) presenting the growth parameters i.e., root and shoot length of tomato plants after 45 days.

A pot bioassay was conducted in a greenhouse to evaluate the in-vivo effectiveness of PP-AgNPs against *F. oxysporum*, which causes tomato wilt (Fig. 4). Table 1 showed a significant reduction in disease attributes (incidence and severity) and improvement in plant growth parameters (root, shoot, and plant height) after treatment with varying concentrations (50–150 µg/mL) of PP-AgNPs. The key features of enhanced retention and permeability make AgNPs intriguing and interesting for distinct antifungal properties^78^. The results indicated that PP-AgNPs at concentrations ranging from 50 to 250 µg/mL significantly decreased the incidence and severity of disease by less than 50% compared to the control treatment. In contrast to the control treatment, which had a 100% incidence of disease, the PP-AgNPs (at concentrations of 50–250 µg/mL) showed a decreasing trend of 49.6%, 32.3%, 34.8%, and 35.9%, respectively. Similarly, the frequency and severity of the disease also showed a steady trend. Infected tomato plants exposed to PP-AgNPs at different concentrations had a disease severity of 45.5%, 34.7%, 34.5%, and 35.1%, respectively, in contrast to the control treatment (96.6%). The antimicrobial mechanism of silver NPs against plant pathogens involves abolition or impedance to pathogen growth without deteriorating the adjacent plant tissues^81^.

**Table 1:** Effect of PP-AgNPs on growth variables and disease attributes in tomatoes infected with Fusarium wilt under greenhouse conditions.

Treatment	Disease incidence (%)	Disease severity (%)	Plant height (cm)	Total length (cm)		Total biomass (g)	
				Root shoot		Fresh dry	
Control	100^a^ (± 0.02)	96.6^a^ (± 3.34)	27.5f. (± 0.19)	12.7f. (± 0.19)	14.8^e^ (± 0.18)	24.8^e^ (± 0.21)	2.34^c^ (± 0.05)
Fungicide	43.3^c^ (± 0.22)	38.7^bc^ (± 2.02)	41.6^d^ (± 0.29)	19.5^d^ (± 0.16)	22.1^d^ (± 0.14)	41.7^c^ (± 0.19)	3.98^b^ (± 0.04)
PP-AgNPs-50 µg/mL	49.6^b^ (± 0.14)	45.5^b^ (± 2.94)	40.4^e^ (± 0.28)	18.8^e^ (± 0.23)	21.6^d^ (± 0.18)	37.3^d^ (± 0.14)	4.08^ab^ (± 0.07)
PP-AgNPs-100 µg/mL	32.3^f^ (± 0.39)	34.7^c^ (± 3.73)	50.6^a^ (± 0.26)	22.1^a^ (± 0.17)	28.5^a^ (± 0.11)	45.9^a^ (± 0.20)	4.24^a^ (± 0.03)
PP-AgNPs-125 µg/mL	34.8^e^ (± 0.23)	34.5^c^ (± 3.47)	47.9^b^ (± 0.28)	21.2^b^ (± 0.12)	26.7^b^ (± 0.21)	43.7^b^ (± 0.15)	4.17^a^ (± 0.04)
PP-AgNPs-150 µg/mL	35.9^d^ (± 0.18)	35.1^c^ (± 3.43)	44.7^c^ (± 0.23)	20.3^c^ (± 0.21)	24.4^c^ (± 0.25)	41.2^c^ (± 0.16)	4.12^ab^ (± 0.08)
CV (%)	**0.80**	**11.65**	**1.06**	**1.67**	**1.39**	**0.79**	**2.41**

Different letters per column indicate significant differences (*P* ≤ 0.05) among treatments, determined by the LSD Fisher test. Each value of data is the average of three replicates. Values with ± indicate standard error between the mean of different replicates of the same treatment. CV represents the coefficient of variation.

Silver nanoparticles emerge as the most captivating metal NPs that reveal adequate bioactivities. At certain concentrations, they prevent the growth of pathogenic fungi^82^ by imparting a wide range of promising effects on plant growth and development^83^. Some earlier studies reported that AgNPs improved germination by enhancing vegetative growth^84^, shoot initiation, pigmentation, and proliferation^85,86^. Applying PP-AgNPs at a 100 µg/mL concentration resulted in the greatest improvement in growth parameters compared to the control and other treatments. Plant height increased gradually at all PP-AgNP concentrations (40.4, 50.6, 47.9, and 44.7 cm), with the highest increase observed at 100 µg/mL. The plant height was superior to the control by 46.9%, 84%, 74.2%, and 62.5%, respectively.

Moreover, when treated with 100 µg/mL, the average length of the roots and shoots of the plants increased by 74% and 92.6%, respectively, compared to the control treatment. The length of the roots and shoots was 22.1 cm and 28.5 cm, respectively (Fig. 4B,C-d). Additionally, the fresh and dry weights of the plants treated with 100 µg/mL were significantly higher than those of the control, increasing by 85.5% and 81.2%, respectively. The application of fungicide resulted in a corresponding increase in plant height, root length, and shoot length when compared to the control group. The growth percentages were measured to be 46.9%, 53.3%, and 49.3%, respectively (Fig. 4B,C-b). The application of silver nanoparticles (AgNPs) has been found to positively impact preventing the infection of *Puccinia striiformis*, *Fusarium oxysporum*, and *Verticillium dahlia* in wheat, tomato, and eggplant. Various studies have shown that using AgNPs to combat these plant diseases can lead to higher crop yields^10,87,88^. Likewise, the findings of Li et al.^68^ suggested that AgNPs act as antifungal alternatives to hinder the growth and development of kiwifruit rot pathogens and are potentially suitable for horticultural crops that directly produce fruits.

### Effect of PP-AgNPs on fruit variables and bioactive-compounds

Tomato fruit is cogitated as a highly nutritious and beneficial food for human health due to rich in bioactive compounds and high antioxidant activity^89^. After being treated with different concentrations of PP-AgNPs, the fruit variables, including weight and number, were significantly affected, as shown in Table S2* and Fig. 4B. The average fruit weight significantly increased to 50.2% at a 100 µg/mL concentration compared to the control group. Additionally, the same concentration of PP-AgNPs resulted in the highest number of fruits, with an increase of 55.4% (33.5 fruits per plant). Conversely, the fungicide treatment also showed a similar trend, with a 39.9% increase in fruit per plant, yielding 30.1 fruits per plant. Some previous reports also indicated the positive effects of other types of nanoparticles on tomato yield. For example, biomass yield increased considerably by 70% compared to the untreated control tomato fruit after applying TiO_2_ nanoparticles @ 250 mg/kg^90^. Furthermore, a significant increase in tomato yield and biomass was reported^91^ after foliar application of CuO nanoparticles under greenhouse and field conditions.

The treatment with varying concentrations of PP-AgNPs significantly enhanced the production of bioactive compounds in tomato fruits from infected plants (Table S2). Lycopene is a strong natural antioxidant and the main carotene available in ripe tomato fruit; this biomolecule, along with $\beta$-carotene, succors in neutralizing peroxyl radicals due to its higher antioxidant capacity and plays a very important role in human health^92^. Compared to untreated control fruits, the lycopene content of treated diseased fruits increased significantly (*p* ≤ 0.05) by 77.8% at 100 µg/mL. Our findings align with the earlier work, which found that, relative to the untreated control, the treatment of nanoparticles increased the lycopene content in tomato fruit by 80% (TiO_2_) and 113% (ZnO)^90^. Under stress, plants respond naturally by involving the synthesis of non-enzymatic antioxidant compounds, i.e., flavonoids and Vitamin C^93^. A similar trend was observed for flavonoid content at all concentrations (50–150 µg/mL), exceeding the control by 36.2–53.8% respectively. The antioxidant defense system of plants is lethally affected after the attack of pathogens; selenium NPs increased the synthesis of flavonoid compounds in malformed infected mango in contrast to untreated infected plants^27^. Vitamin C improved with all applied treatments in a range of 16.1–24.8% compared to the untreated control. The most effective treatment, however, was 100 µg/mL, significantly increasing over the control by 81.4%. According to Srichiangsa^94^, mature tomatoes treated with silver-decorated magnetic particles (Ag@Fe_3_O_4_) had higher vitamin C contents during storage. Moreover, total protein content (> 50%) was also upregulated in response to silver NPs.

### Effect of PP-AgNPs on enzymatic compounds

Plants can produce antioxidant enzymes that help to mitigate the harmful effects of reactive oxygen species (ROS). Environmental stressors can negatively impact a plant’s electron transport system and contribute to the production of ROS^27^. The Figs. S2 and S3 display the activity of antioxidant enzymes (POD, SOD, CAT, PAL, and PPO) in the roots and shoots of tomato plants treated with PP-AgNPs. Antioxidant enzymes dispense an increased level of defense interconnected with Fusarium infection that acts as an initial stage in enhancing a plant’s resistance against different stresses^95^. Figure S2A displays the POD enzyme activity in tomato plant roots and shoots treated with varying concentrations of PP-AgNPs. The results indicate that 100 µg/mL of PP-AgNPs caused the highest activity compared to the control. This concentration caused roots and shoots to increase by 1.94 and 1.87 fold, respectively. A similar upsurge was detected for SOD activity in PP-AgNPs treated tomato plants (Figure S2B). All concentrations significantly improved root and shoot growth by 2.52–3.09 and 2.63–2.82-fold respectively, compared to control. A study by Zahedi et al.^96^ also notices enhanced activities of POD and SOD in stressed plants after treatment with nanoparticles. Conversely, substantial catalase production was estimated, and the best treatment was 100 µg/mL, superior to the control by a fold of 1.88 in roots and 1.84 in shoots (Figure S2C). Our findings regarding POD, SOD, and CAT activities are in line with the findings of Karami Mehrian et al.^97^, who reported increased activity of antioxidant enzymes in tomato plants (roots & shoots) after treatment with silver NPs.

PAL has been a widely accepted biomarker involved in activating plant defense mechanisms under a stressful environment; synthesis of phenylpropanoid phytoalexins occurs in response to pathogen attacks and incites the rapid initiation of PAL enzyme^92,98^. The PAL activity was highest at 100 µg/mL of PP-AgNPs (Figure S3A) across all treatments. It was 2.57 and 2.35 times greater than the control in roots and shoots. In addition, roots and shoots treated with fungicide outperformed the control by 2.06 and 1.87 times, respectively. This suggests that the increased resistance against *F. oxysporum* may be due to the elevated PAL activity that results from the application of PP-AgNPs. Previous studies have shown similar results when using silver nanoparticles in the treatment of various plant diseases^99,100^.

The PPO enzymes support the plant defense system by providing a physical impediment against many phytopathogens involved in lignin biosynthesis, reducing the negative impact of stresses by scavenging reactive oxygen species through fungal pathogens through phenolic compounds^101,102^. Figure S3B illustrates the production of PPO in tomato plant roots and shoots after PP-AgNP treatment. The most significant activity was observed at a concentration of 100 µg/mL, where roots and shoots exhibited 2.08–1.99 times higher production of PPO, respectively, compared to the control. Similar results were achieved by Elatafi and Fang^103^, who noticed an increase in PPO activity in grapes after the application of 100 µg/mL of AgNPs.

### Analysis of gene-expression in tomato plants enthused by PP-AgNPs

In comparison to the control group, the analysis using quantitative real-time PCR (qPCR) showed a significant and immediate increase in the expression of defense genes (PAL, PPO, POD, CAT, and SOD) and pathogenesis-related protein genes (PR2 and PR5) in both the roots and shoots of tomato plants.

**Fig. 5:**
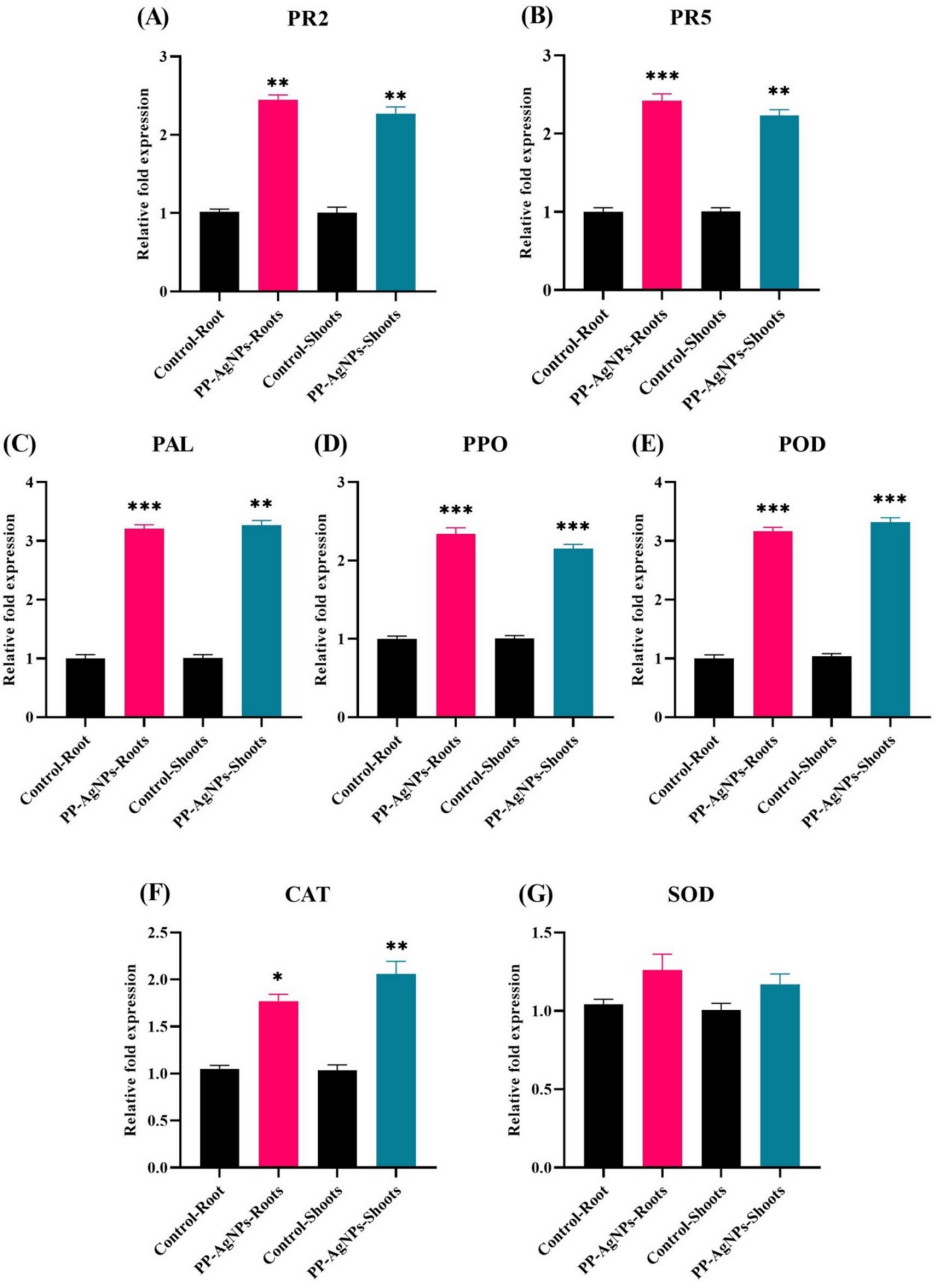
Relative expression of pathogenesis-related protein genes and defense genes in tomato roots and shoots after exposure to PP-AgNPs (100 µg/mL). Data presented as a mean ± SD of replicates showing substantial difference (*p < 0.05, **p < 0.01, ***p < 0.001) in parallel to the control by One-Way-ANOVA (*p* < 0.05) and Tukey’s-multiple comparison analysis using Graph-pad prism.

Under stress, plants develop highly intricate responses that lead to certain alterations to the physiological, cellular, and transcriptomic processes and complex associations between respective signaling pathways generated under adverse stress conditions^104–106^. After treating tomato plants (roots and shoots) with 100 µg/mL of PP-AgNPs, a significant change in the gene expression levels was observed, as measured by qRT-PCR, according to the findings presented in Fig. 5. The expressions of the PR1, PR2, and PR5 genes suggested the activation of the salicylic acid (SA) signaling pathway, which in turn induced the defense mechanism^107^. Upon exposure to PP-AgNPs, there was a notable increase in the relative expression of PR2. As compared to the control, both roots and shoots showed fold increases of 2.45 and 2.27, respectively (as depicted in Fig. 5A).

The PR-5 is a thaumatin-like protein that behaves like an acute defensive gene, upregulated against several phytopathogens^108^. Compared to the control group, the expression level of the PR5 gene in roots showed a significant increase of about 1.96-fold in Fig. 5B, and shoots exhibited an increase of approximately 2.23-fold. Additionally, the roots displayed higher levels of expression for both pathogenesis-related genes than the shoots. Biosynthesized AgNPs activate the immune defense in *Nicotiana benthamiana* by exhibiting a significant upregulation of pathogenesis-related genes 1 and 2^109^. Correspondingly, the earlier findings^110^ also suggested that biosynthesized silver nanoparticles act as elicitors, significantly inducing expression levels of pathogenesis-related genes (PR1 and PR5) in infected squash plants.

The plant defense system is escorted by antioxidant enzymes that play an important role in ROS quenching; therefore, overexpression of antioxidant genes is assumed to be a defense role against ROS generation by silver ions. As stated by Maleki et al.^111^, there is evidence that antioxidative activity stimulates the phenylpropanoid pathway. Earlier studies have shown that up-regulation of the PAL gene promotes the biosynthesis of secondary metabolites, which is beneficial to improving plant defense under stress conditions^108,112,113^. The results presented in Fig. 5C show a significant increase in PAL expression, up to 3.21 times in roots and 3.27 times in shoots compared to the control. These findings are consistent with Noori et al.^114^ who also observed a significant increase in PAL gene expression in tomato plants exposed to 30 mg/L AgNPs. Additionally, the foliar application of AgNPs significantly expresses the PPO gene, surpassing the control by 2.15 and 2.34 times in shoots and roots, respectively (Fig. 5D).

In a study conducted, researchers treated tomato plants infected with nematodes using green synthesized AgNPs. The results indicated a significant improvement in the plant’s immune system, as shown by the increased expression of PAL, PPO, and POD enzymes^115^. Additionally, in tomato shoots exposed to PP-AgNPs, the gene expression of POD and CAT was significantly upregulated by 3.32 and 2.06-fold, respectively, compared to the untreated control (Fig. 5E,F). Gupta and his colleagues observed that when rice seedlings were exposed to varying concentrations of AgNPs, the expression of the SOD gene decreased. In contrast, the CAT and APX genes were significantly up-regulated^116^. Soliman et al.^117^ found that exposure to AgNPs increased antioxidant gene expression in certain plant species. However, compared to the control group, the SOD gene did not show significant expression in either the roots (1.26-fold) or the shoots (1.17-fold) (Fig. 5G). Therefore, the present study suggests that nanoparticles may make tomato plants more resistant to infections. Additionally, the data indicates a positive correlation between the relative gene expression of nanoparticles and their enzymatic activities.

### Field efficacy of PP-AgNPs on enhancing tomato growth variables and yield

The efficiency of the best concentration (100 µg/mL) from PP-AgNPs (T_3_) was explored for the field studies for two consecutive years (Figure S4). Very few studies have explored the potential of nanoparticles in field conditions (soil pH 7–8.5; average temperature 10–32 °C; average humidity 57%). However, our research has shown that nanoparticles can significantly reduce disease severity and improve plant growth parameters compared to untreated plants or those treated with fungicides. Nanoparticles in plant disease management focus on their potential as biostatic agents, nano-pesticides to combat phyto-diseases, and nano-fertilizers to provide micro and macronutrients for plant growth^118^.

In field conditions, Table 2 displays the impact of PP-AgNPs (T_3_) on tomato plant growth variables and yield. The height of the tomato plants significantly increased (*p* < 0.05) in both 2021 and 2022, surpassing the control by 56.6% and 62.6%, respectively. However, the T_2_ (fungicide) treatment resulted in the lowest plant height, with an average increase of 40.9% over the control in both seasons. Additionally, a consistent pattern was observed in the biomass of contaminated tomato plants. In 2022, the fresh and dry biomass exceeded the control by 78.1% and 71.2%, respectively, while in 2021, they outpaced it by 63.7% and 60.1%, respectively. Compared to the control, the application of fungicide (T_2_) resulted in a significant increase in both fresh and dry biomass. However, this increase was lower than the increase observed when using 100 µg/mL of PP-AgNPs. As per Table 2, the estimated average increase in fresh and dry biomass for the years 2021 and 2022 with T_2_ treatment was 39.63% and 35.5%, respectively.

**Table 2:** Effect of PP-AgNPs on growth variables, fruit number, and disease severity in tomatoes infected with fusarium wilt under field conditions during 2021 and 2022.

Treatment	Year-2021						Year-2022					
	Plant height (cm)	Biomass (g)		Disease severity (%)	Fruit number	Yield (kg)	Plant height (cm)	Biomass (g)		Disease severity (%)	Fruit number	Yield (kg)
		Fresh	Dry					Fresh	Dry			
T_1_	86.53^c^ (± 3.86)	90.9^c^ (± 3.19)	13.3^c^ (± 0.45)	67.1^a^ (± 0.20)	18.4^c^ (± 0.26)	4.38^c^ (± 0.22)	83.8^c^ (± 1.25)	87.4^c^ (± 0.59)	12.6^c^ (± 0.25)	68.0^a^ (± 0.23)	19.3^c^ (± 0.19)	4.44^c^ (± 0.24)
T_2_	120.2^b^ (± 3.33)	123.2^b^ (± 4.08)	18.4^b^ (± 0.39)	39.2^b^ (± 0.15)	25.1^b^ (± 0.18)	5.32^b^ (± 0.24)	119.8^b^ (± 1.88)	125.6^b^ (± 1.00)	16.7^b^ (± 0.34)	37.5^b^ (± 0.30)	23.7^b^ (± 0.24)	5.73^b^ (± 0.22)
T_3_	135.5^a^ (± 3.10)	148.8^a^ (± 3.70)	21.3^a^ (± 0.18)	23.8^c^ (± 0.16)	31.5^a^ (± 0.26)	6.21^a^ (± 0.22)	136.2^a^ (± 2.25)	155.7^a^ (± 1.59)	21.5^a^ (± 0.18)	22.2^c^ (± 0.21)	34.5^a^ (± 0.27)	6.58^a^ (± 0.27)
CV (%)	**5.20**	**5.23**	**3.38**	**0.68**	**1.62**	**7.41**	**2.73**	**1.49**	**3.35**	**1.01**	**1.56**	**7.61**

*Growth parameters and disease severity was recorded after 1 month of transplantation in the field whereas fruit number and yield were noted after 3 months. T_1_ = Positive control (*Fusarium oxysporum* only); T_2_ = Negative control (Fungicide + *Fusarium oxysporum*); T_3_ = 100 µg/mL of PP- AgNPs + *Fusarium oxysporum*. Different letters per column indicate significant differences among treatments, determined by ANOVA and LSD-test at (*P* ≤ 0.05). Values with ± indicate standard error between mean of different replicates of same treatment. CV represents the coefficient of variation.

Our results align with the work of Ansari et al.^119^ found a significant increase in the height, fresh weight, and dry weight of tomato plants treated with green-synthesized silver nanoparticles compared to the control group. Various concentrations of AgNPs promoted the growth and flowering of oriental lilies^16^. However, the chemical composition and absorbance intensity of the leaves treated with AgNPs did not change, according to FTIR spectra. Similarly, AgNPs stimulated rice plants’ growth, resulting in longer and higher biomass seedlings^116^. In addition to enhancing tomato plant resistance against fusarium wilt infection, nanoparticle treatments also increased the fruit yield and its quantity.

The results showed that using PP-AgNPs significantly increased the yield and fruit count (Table 2). When compared to the control group, there was a 70.8% and 79% increase in fruit number in both years, respectively. However, the fungicide treatment (T_2_) resulted in a lower fruit count, with an average of only 29.6% in both seasons. Additionally, five plants were randomly selected after the final harvest to examine the yield following treatment with PP-AgNPs. The maximum yield observed in T_3_ (PP-AgNPs) showed a significant increase in both 2021 and 2022, with a rise of 41.8% and 48.2%, respectively, over the control group (T_1_). Table 2 indicates that the lowest yield was observed in treatment T_2_ (fungicide) during both seasons, surpassing the control by 21.4% and 29.1%, respectively.

After receiving treatment with AgNPs, two different tomato plant varieties (Nadar and Naqeeb) showed an increase in their yield (ranging from 35 to 43%) and the number of tomato fruits per plant (ranging from 20 to 36 and 21 to 37)^119^. The application of 100 µg/mL of PP-AgNPs (T_3_) and fungicide (T_2_) led to a significant reduction in disease severity in infected tomato plants as compared to the control group. The results in Table 2 demonstrate that T3 treatment exhibited the lowest disease index with suppressive activity of 64.5% and 67.4% in 2021 and 2022, respectively. In contrast, fungicide (T_2_) resulted in a suppressive activity of 44.9% in 2022 and 41.7% in 2021.

Research conducted by Alvarez-Carvajal and colleagues found that after 14 days post-inoculation, the use of silver nanoparticles coated with chitosan resulted in a dramatic reduction (> 70%) of mycelial growth in tomato plants infected with *F. oxysporum*. Importantly, this treatment did not have any discernible detrimental effects on the vegetative growth of the tomato seedlings^120^. Correspondingly, Akpinar et al.^121^ suggested that using AgNPs may also be effective in impeding the growth of *F. oxysporum* f. sp. *lycopersici* in tomato plants, which could make them a promising protective agent for promoting tomato growth.

### Silver (Ag) concentration in tomato plants after exposure to PP-AgNPs

Metal accumulation and uptake in the tomato plant’s tissues and fruit were examined using an atomic absorption spectrophotometer (AAS). It is plausible to assume that the inhibition of the fungal disease may be directly correlated with an increase in metal content. After 2 years of exposure to the best treatment of PP-AgNPs (T_3_) in the field, significant accumulation and uptake of metal content in the roots, shoots, and fruits of tomato plants were observed in 2021–2022, as shown in Table S3.

In 2021, plants that were exposed to 100 µg/mL of PP-AgNPs (T_3_) had significantly higher levels of Ag metal. The roots, shoots, and fruits had 12.9, 24.8, and 2.29 times higher Ag metal, respectively, as compared to the control plants (T_1_). In 2022, tomato plants infected with fungal disease were found to have significantly higher Ag content in their roots, shoots, and fruits. The Ag content in the roots, shoots, and fruits was 11.9, 24.2, and twice as high as the control. It can be assumed that the increase in metal content directly correlates with inhibiting the fungal disease. The tomato fruits were found to have the lowest metal concentration, indicating that they are safe to eat, while the roots had the highest metal content. A study was conducted on tomato roots exposed to different ionic silver and silver nanoparticles (AgNPs) concentrations showed increased Ag content ranging from 433 to 71 µg/mL for ionic silver and 40–47 µg/mL for AgNPs^122^. Similarly, Castro-Gonzalez^123^ discovered that a topical application of AgNPs (100 and 200 mg L^−1^) to stevia plants resulted in an accumulation of Ag content in the shoots, measured at 95.23 and 188.16 µg/g dw. Geisler-Lee et al.^124^ found that the foliar application of AgNPs, *Arabidopsis thaliana* root, and shoot tissue showed ten times higher Ag content in roots than shoots.

According to the EPA, the acceptable reference dose (RfD) for silver exposure is 5 µg per kilogram of body weight per day. EFSA indicates no adverse effect level for silver in human diets, meaning that daily intake of silver from food should not exceed. 0.005 mg/kg of body weight^125,126^. Silver residues in crops should be minimal. Levels above 0.1 mg/kg (100 µg/kg) in the edible parts of plants, such as fruits, are typically marked for further investigation to evaluate safety. Although we used silver in the form of nanoparticles, the amount of metal content in the main product, tomato fruit, is below the recommended doses. However, future studies on model animals, such as rats or rabbits, are still recommended to assess silver nanoparticles’ effects on various animal organs.

## Conclusions

The successful development of PP-AgNPs through a green and sustainable synthesis approach offers a promising solution to combat *Fusarium oxysporum*, a notorious soil-borne pathogen responsible for causing severe wilt in tomato plants. By inducing the generation of reactive oxygen species (ROS) at the fungal cell wall, PP-AgNPs employ a powerful mechanism to destroy fungal cells, paving the way for enhanced pathogen control strategies. In both greenhouse and field trials, applying PP-AgNPs to infected tomato plants markedly reduced disease severity while enhancing crop yield and productivity. Notably, treated plants exhibited remarkable improvements in growth parameters, including increased plant height, biomass, fruit weight, fruit count, and enriched bioactive compounds.

Our findings underscore the ability of PP-AgNPs to activate antioxidative and defense responses at both molecular and physiological levels in plants. Importantly, these nanoscale agents showed no visible signs of toxicity, ensuring safe and effective integration into agricultural practices. This innovative solution offers a sustainable pathway to combat plant pathogens and boost crop productivity, making it a promising tool for addressing global food security challenges.

While the current study demonstrated the effectiveness against *F. oxysporum* in tomatoes, future research should investigate long-term impact assessments to ensure environmental safety and stability. Furthermore, expanding the scope of these nanoscale agents across diverse crop-pathogen systems could unlock their broader agricultural potential. By integrating PP-AgNPs into various agronomic frameworks, researchers can explore their adaptability, scalability, and synergistic effects with other sustainable practices. Such studies could provide critical insights into their role in fostering resilient crop production systems, ultimately contributing to global food security in the face of climate change and escalating agricultural demands. PP-AgNPs stand poised to become a cornerstone in the next generation of agro-nanotechnological advancements.

## Supplementary Information

Supplementary Information.

## Data Availability

All data generated or analysed during this study are included in this published article and its supplementary information files.
