# Supplementary material for: Phytofabricated silver nanoparticles unlock new potential in tomato plants by combating wilt infection and enhancing plant growth
Source: Sci Rep. 2025 Mar 27;15:10527. doi: 10.1038/s41598-025-89724-4 (PMC11950516; doi:10.1038/s41598-025-89724-4)
Supplement: Supplementary file 1 — Supplementary Information. [file 41598_2025_89724_MOESM1_ESM.docx]

**Supplementary Information**

**Phytofabricated Silver Nanoparticles Unlock New Potential in Tomato Plants by Combating Wilt Infection and Enhancing Plant Growth**

Hina Ashraf ^(a, c, f) *^, Tehmina Anjum^(a)^, Irfan S. Ahmad ^(b, c)^, Rashid Ahmed^(d)^, Zill-e-Huma Aftab^(a)^, Humaira Rizwana ^(e)^

(a) Department of Plant Pathology, Faculty of Agricultural-Sciences, University of the Punjab, Lahore, Pakistan

(b) Department of Agricultural & Biological Engineering, University of Illinois at Urbana- Champaign, IL, USA

(c) Holonyak Micro and Nanotechnology Laboratory, University of Illinois at Urbana- Champaign, IL, USA

(d) Department of Biotechnology, Mirpur University of Science and Technology (MUST), Azad Jammu and Kashmir, Pakistan

(e) Department of Botany and Microbiology, College of Science, King Saud University, Riyadh, Kingdom of Saudi Arabia

(f) Department of Analytical Chemistry, The Connecticut Agricultural Experiment Station, New Haven, Connecticut, USA

***Correspondence:**

**Email:** [**Hina.Ashraf@ct.gov**](http://Hina.Ashraf@ct.gov)

***Experiment S1. Characterization of green-synthesized PP-AgNPs***

The green synthesis of PP-AgNPs was optimized by adjusting reaction parameters, employing a Denovix DS-C UV-visible spectrophotometer with a wavelength range of 220-720 nm and a 1 nm resolution. Following synthesis, the nanoparticles (NPs) were further purified by undergoing multiple rounds of centrifugation at 6000 rpm for 30 minutes at 4°C. The supernatant produced in this process was discarded, and the resulting pellets were allowed to air-dry at 60°C for 24 hours. Subsequently, the oven-dried PP-AgNPs were subjected to various analytical techniques to ascertain their specific characteristics. The functional characteristics of nanoparticles were investigated using Fourier transform infrared spectroscopy (FTIR-Thermo Scientific Nicolet 6700), covering a spectral range of 400-4000 cm^-1^, with a resolution of 4 cm^-1^. To assess the crystallographic arrangement of biosynthesized nanoparticles, X-ray diffraction (XRD) patterns were obtained utilizing a Philips PANalytical XPert Powder diffractometer within an angular range of 2θ = 20°- 80°. The nanoparticle size was determined through dynamic light scattering (DLS) to measure diameter and zeta potential. In contrast, size and stability were assessed employing a Zetasizer Nano ZS instrument (Malvern Instruments, UK) at 25 °C, assuming a water refractive index of 1.33. Morphological attributes, size, and elemental composition were ascertained using a variable pressure Scanning Electron Microscope (SEM) equipped with an energy-dispersive X-ray (EDX) instrument (SEM-TESCAN-VegaLMU, 30.0 kV). The Transmission Electron Microscope (TEM- JEOL 2010F) was also employed to capture images illustrating the nanoparticles' average size, morphology, and electron diffraction patterns (SAED).

***Experiment S2. Analyzing morphological variation, ROS induction, cell-wall, and membrane integrity***

To evaluate the deteriorating effect of PP-AgNPs on ultrastructural and plasma membranes, cell-wall integrity and ROS generation in *F. oxysporum* were examined by following the protocols performed in our previous studies ^1^. Briefly, mycelia and spores (centrifugation at 35,000 rpm) of fungus (control and treatment) were pre-fixed with glutaraldehyde (2.4%) for 2 hours at 4 °C and then post-fixed with aqueous osmium tetroxide (OsO_4_, 1%) and later washed with phosphate buffer (0.1 M, pH 7.8). A graded series of ethanol (30, 50, 70, 80, 90, and 100%) was used for dehydration of samples, subsequently, passed through acetone and dried in a vacuum oven to be observed under an SEM (S-4800, Hitachi, Japan)

2.5 µg mL-1 of propidium iodide (PI-Sigma Aldrich) was used to stain mycelia for 15 min at 30 °C under dark conditions to analyze the membrane permeability. Likewise, cell-wall integrity in treated and control samples was observed after staining with 2 μL of 10 μg/mL of CFW for 30 min at room temperature under dark conditions. Moreover, 2 μL of 2′,7′ dichlorofluorescin diacetate (DCFH-DA-Sigma Aldrich) (30 μM) was added to the hyphal suspension and incubated for an hour under dark conditions at room temperature for proper staining. For the control sample, the hyphae were treated with sterilized distilled water. The images of the samples were captured using an upright fluorescence microscope (DM,

Leica Germany).

***Experiment S3. Quantification of stress enzymes***

Stress enzymes in tomato plants (roots and shoots) were determined on the 5^th^ day after treatment with different concentrations of PP-AgNPs. The guaiacol colorimetric method measured the peroxidase activity (POD) at 470 nm. Catalase (CAT) activity was measured at 240 nm using the Cakmack and Horst ^2^ method, which was based on the rate of oxidation of H_2_O_2_. The method developed by Dhindsa et al. ^3^ to measure the ability of superoxide-dismutase (SOD) to impede the photochemical reduction of nitro blue tetrazolium (NBT) at 560 nm was used to measure SOD activity. Using catechol as a substrate at 495 nm, polyphenol oxidase (PPO) activity was measured using a method described by Cheema and Sommerhalter ^4^. Using trichloroacetic acid at 290 nm, phenylalanine ammonia-lyase (PAL) activity was measured according to Syklowska-Baranek et al.^5^

***Experiment S4. Impact of PP-AgNPs on the regulation of gene expression in tomato***

Gene-expression studies were performed to elucidate the effect of PP-AgNPs on tomato plants (roots and shoots). qRT-PCR was employed to evaluate the alteration in transcript-level of pathogenicity (PR2 and PR5) and defense-related genes (PPO, PAL, POD, CAT, SOD) after exposure to 100 µg/mL of PP-AgNPs. The Protocol was used with some minor amendments for isolation of total RNA, synthesis of cDNA, and quantification by qRT-PCR. Briefly, 100 mg of lyophilized root and shoot samples were ground using liquid nitrogen to extract total RNA. The Ribosin^TM^ plant kit (Gene All, Seoul, Korea) was used for the extraction process, following the manufacturer's instructions. The concentration of RNA was measured using Nanodrop spectrophotometry (NanoDrop 1000, Thermo Scientific, USA). To synthesize complementary DNA (cDNA), the purified RNA (stored at -20 °C) was processed by using the GScript First-Strand Synthesis Kit (GeneDireX, New Taipei, Taiwan) as per the kit's instructions. The primer specificities of RT-PCR products were confirmed by performing agarose gel electrophoresis. For the qRT-PCR analysis, the PikoRealTM Real-Time 96 PCR System (Thermo Scientific, Waltham, MA, USA) was utilized. The amplification program for all reactions was adjusted by following the initial denaturing step of 95 °C for 10 minutes (optics-off), a loop of 40 cycles at 95 °C for 15 seconds (optics-off) and 60 °C for 30 seconds (optics-on), and a temperature ramp from 60 °C to 95 °C at 0.2 C/s for melting-curve analysis. The analysis used a 100 ng/µL cDNA template with Maxima SYBR Green qPCR Master Mix (2X) (Thermo Scientific, USA). The housekeeping gene actin was used, and each qRT-PCR reaction, including the positive control (infected plants treated with nanoparticles) and negative control (infected plants not treated), was amplified in triplicate. The qRT-PCR analysis used a total volume of 20 µL to obtain the threshold-cycle (Ct) value using Thermo Scientific PickoRealTM software 2.1 (Thermo Scientific, Waltham, MA, USA). The final quantification values were analyzed and calculated using the 2-ΔΔCt method, as described by Livak and Schmittgen^6^ to determine the relative level of gene expression in different treatments. The list of primer sequences used in the study is presented in Table S1.

***Experiment S5. Estimating silver content in tomato (roots, shoots, and fruits)***

After the final harvest, tomato plants' fruits, shoots, and roots, which had been treated with PP-AgNPs and the positive control, were washed twice with deionized water to remove any remaining soil particles and dust. The plant samples were then dried in an oven at 60 ºC for 72 hours. Additionally, 100 mg of ground dry samples were digested at 95 ºC in a water bath for an hour using 3 mL of pure nitric acid (65%). After cooling, 0.5mL of 30% H_2_O_2_ was added, and the samples were heated to 100 ºC until no more effervescence was visible. The samples were then allowed to cool and filtered at room temperature. An atomic absorption spectrophotometer (Shimadzu 6800) was used to measure the amount of silver, and the estimated elemental concentration was expressed as µg/g (dry tissue weight).


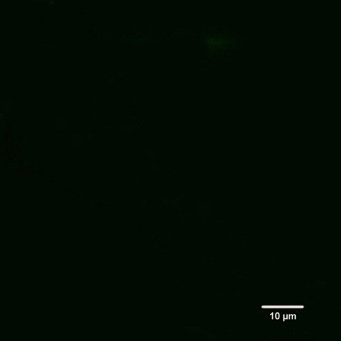

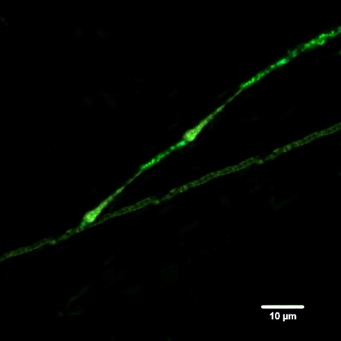

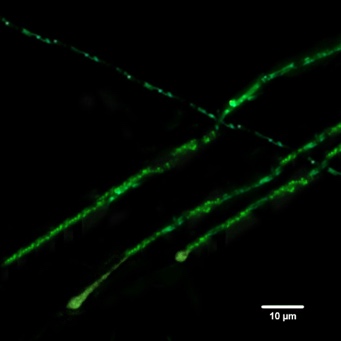

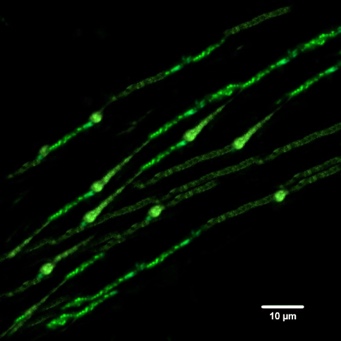

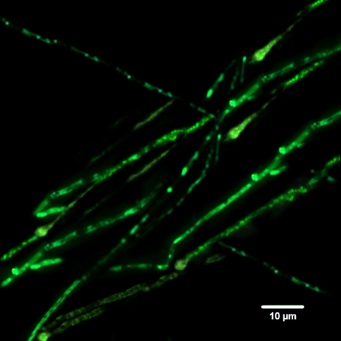

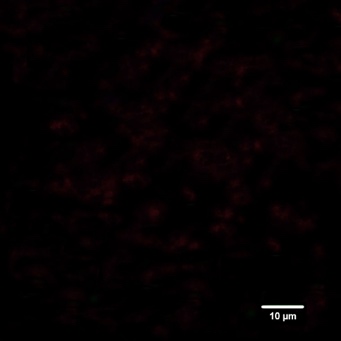

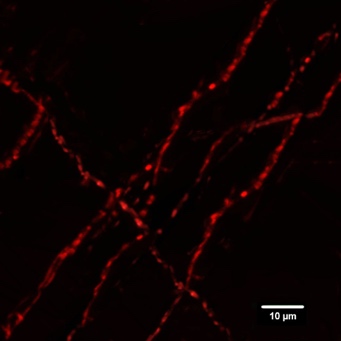

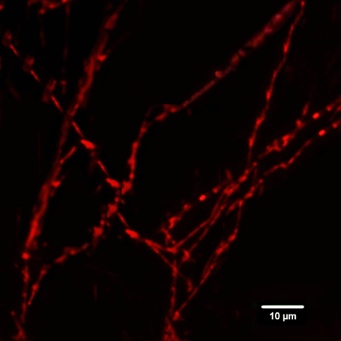

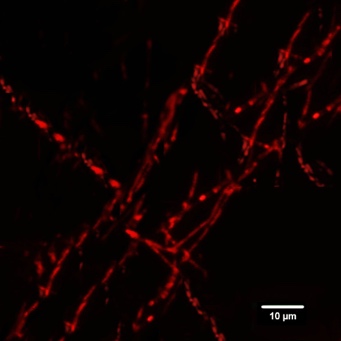

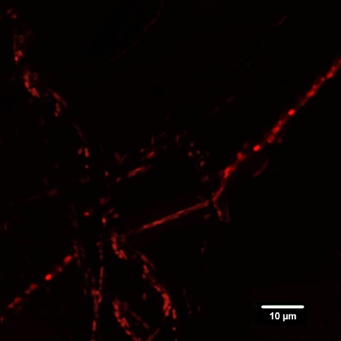

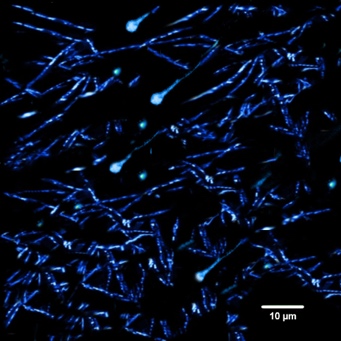

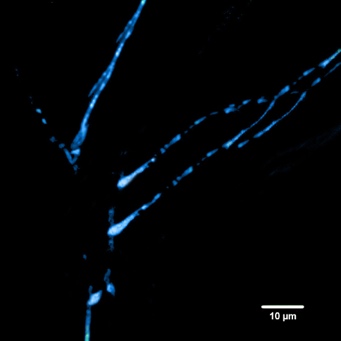

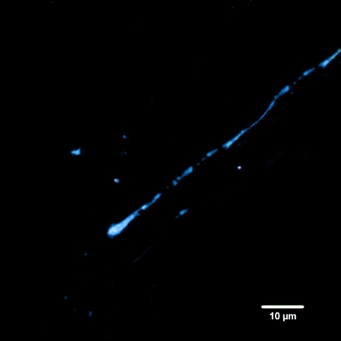

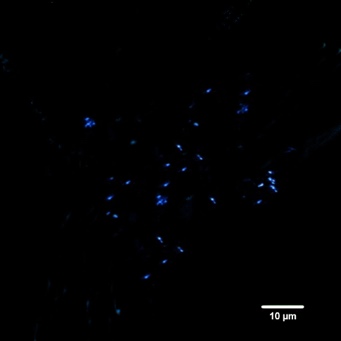

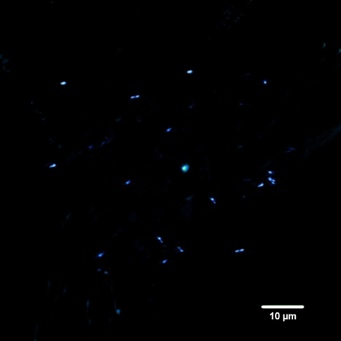


**Control-hyphae of *Fusarium oxysporum***

**PP-AgNPs-50 µg/mL**

**PP-AgNPs-100 µg/mL**

**PP-AgNPs-125 µg/mL**

**PP-AgNPs-150 µg/mL**

**DCFH-DA**

**PI**

**CFW**

**(A)**

**(H)**

**(C)**

**(L)**

**(G)**

**(B)**

**(K)**

**(F)v**

**(M)**

**(D)**

**(I)**

**(N)**

**(E)**

**(J)**

**(O)**

**Figure S1:** Fluorescence microscopy of *F. oxysporum* mycelium treated with different concentrations of PP-AgNPs nanoparticles (50-150 µg/mL) and control (sterile water). **(A-E):** Represents accumulation of reactive oxygen species (ROS) in hyphal cells stained with Dichloro-dihydro-fluorescein diacetate (DCFH-DA); **(F-J):** Fungal hyphal cells with damaged cell-membrane showed red fluorescence stained with Propidium iodide (PI), **(K-O):** Displays fluorescence micrograph of hyphal cells stained with Calcofluor white (CFW) indicating low

glucan and chitin content.


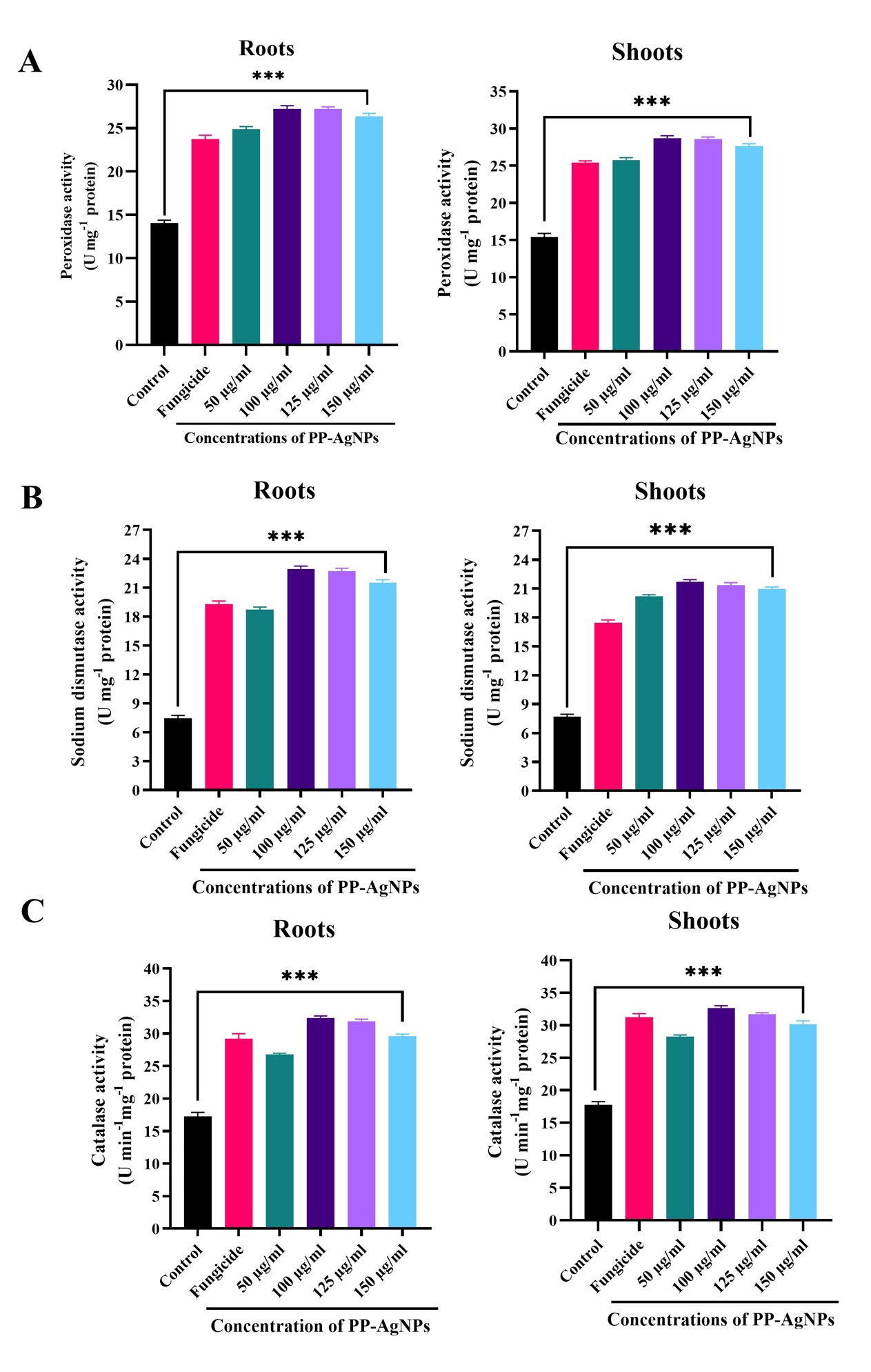


**Figure S2:** Effect of various concentrations PP-AgNPs on enzymatic antioxidant compounds: **A:** POD, **B:** SOD, and C: CAT in the roots and shoots of tomato plants infected with Fusarium oxysporum under greenhouse conditions. Data presented as a mean ± SEM (n=5) of replicates showing substantial difference (*p<0.05, **p<0.01, ***p<0.001: ns: non-significant) in parallel to the control by One-Way-ANOVA (p<0.05) and Tukey’s-multiple comparison analysis using Graph-pad prism.


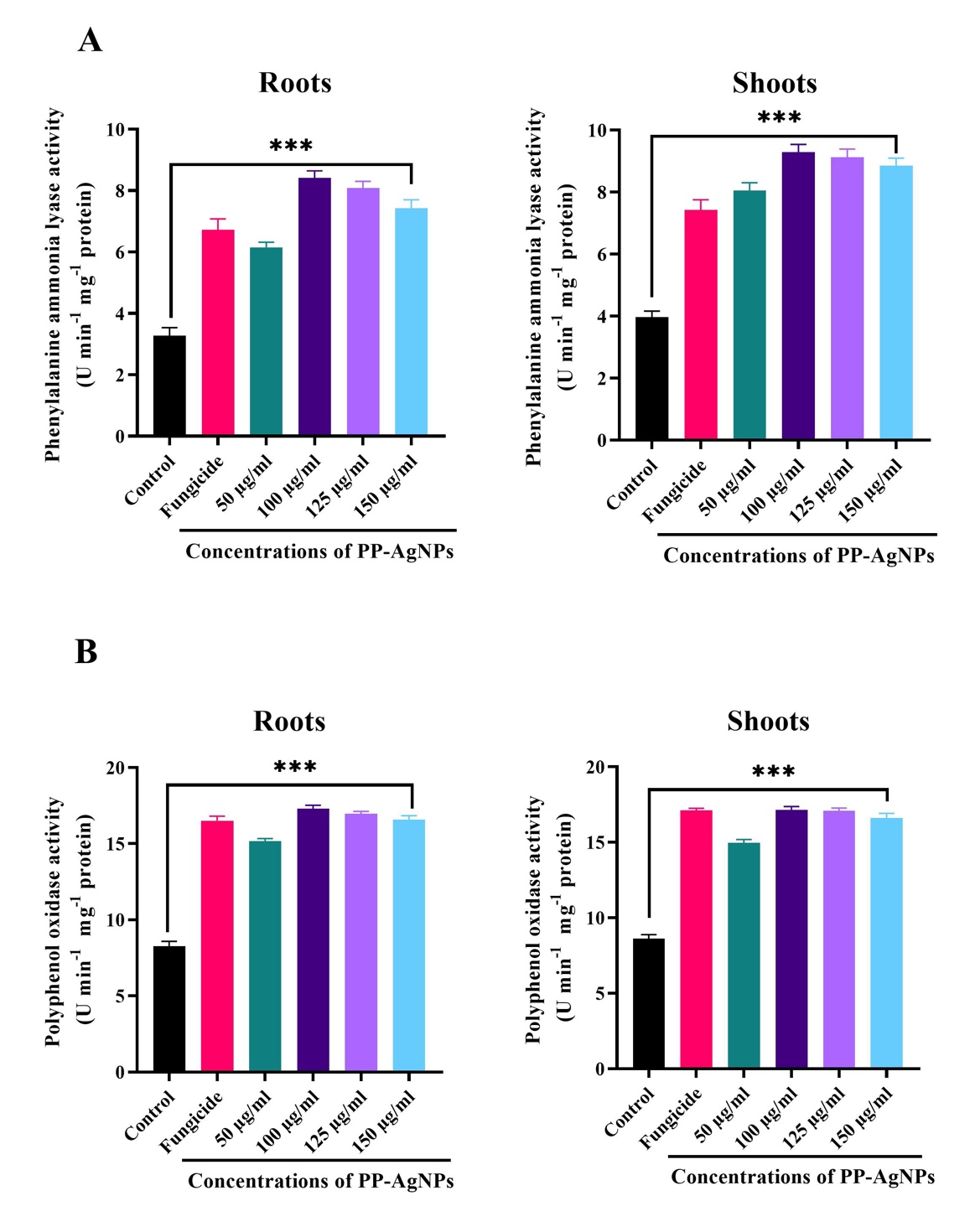


**Figure S3:** Effect of various concentrations PP-AgNPs on stress enzymes: **A:** PAL and **B:** PPO in the roots and shoots of tomato plants infected with *Fusarium oxysporum* under green-house conditions. Data presented as a mean ± SEM (n=5) of replicates showing substantial difference (*p<0.05, **p<0.01, ***p<0.001: ns: non-significant) in parallel to the control by One-Way-ANOVA (p<0.05) and Tukey’s-multiple comparison analysis using Graph-pad prism.


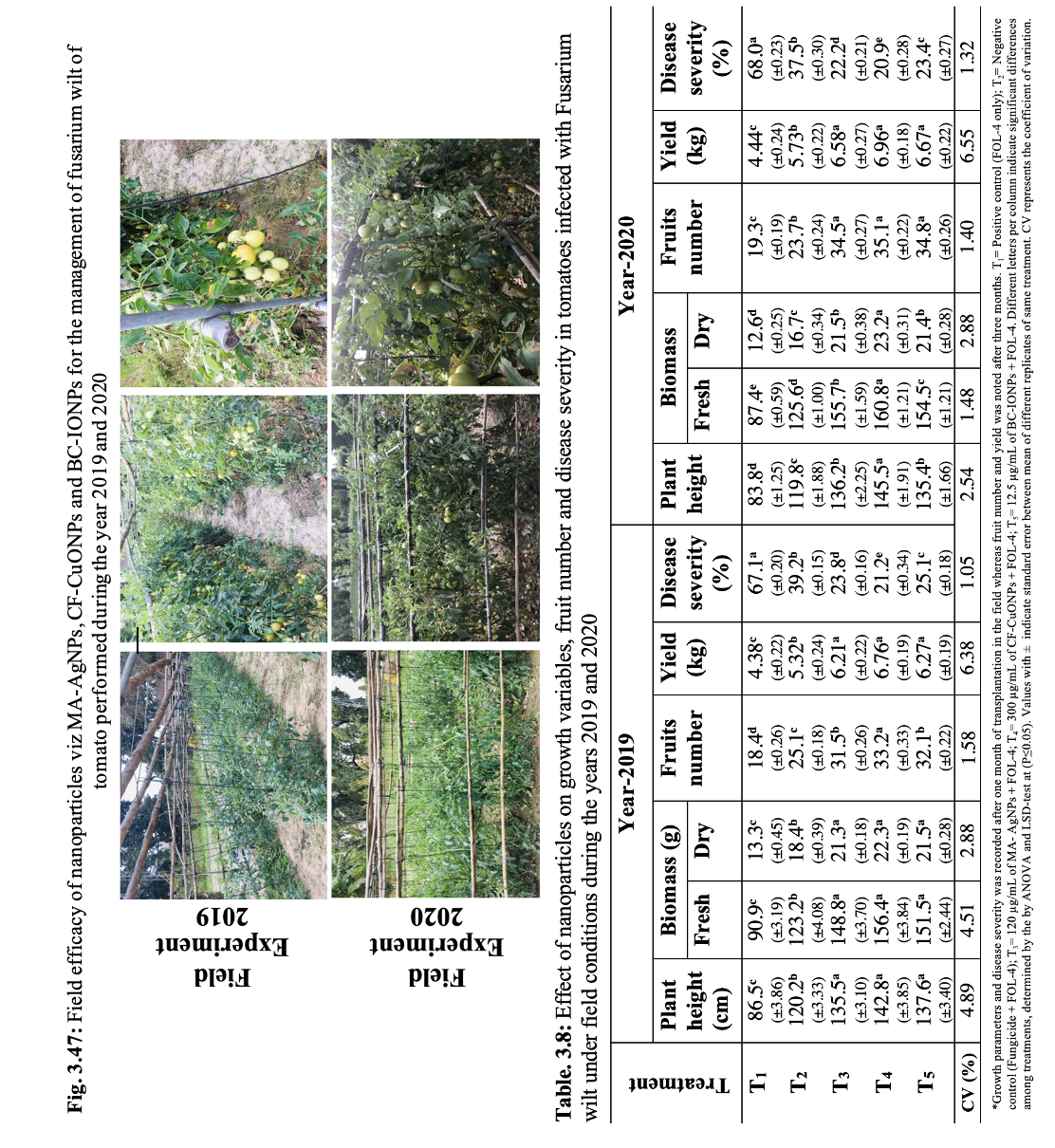


**Field Experiment**

**2021**

**Field Experiment**

**2022**

**Figure S4.** Field efficacy of PP-AgNPs (100 µg/mL) for managing fusarium wilt of tomato during the years 2021 and 2022.

**Table S1.** List of pathogenicity-related and defense-gene primers sequence for qRT-PCR analysis

| Primers | Primer Sequence (5’-3’) |
| --- | --- |
| ACTIN-F | CATTGTGCTCAGTGGTGGTTC |
| ACTIN-R | TCTGCTGGAAGGTGCTAAGTG |
| PR2-F | GTTTACTGCGCTACCTGGGA |
| PR2-R | CCTGTGTTGGTCACCCTCAA |
| PR5-F | GAGGTTCATGCCAAACTGGTC |
| PR5-R | CCGTCAACCAAAGAAATGTCC |
| PAL-F | TTATTAGGTTCTTGAATGCTGGAGT |
| PAL-R | CAAACACGGGGTGATGTTGC |
| PPO-F | CTTCTGTGACTAAGCTCCGTATT |
| PPO-R | AGGGTTATCAGGTTGTGTCTTATC |
| POD-F | ACGGAGCAAGCGACAATTGACAAC |
| POD-R | CGATTGATTCACCGCAAAGCTCGT |
| CAT-R | GCACAGGGATGAGGAGATCG |
| CAT-F | TCTGTCGGGTGTGAATGAGC |
| SOD-R | AAGGGTGACCTGAGACTCTTG |
| SOD-F | GTGAAGAGGATGGTGCCACT |

**F-forward Primer; R-reverse primer**

**Table S2.** Effect of PP-AgNPs on average weight, number, proteins, and non-enzymatic antioxidant compounds in tomato fruits infected with Fusarium wilt under greenhouse conditions.

| **Treatment** | **Average Fruit**  **weight (g)** | **Fruits Number** | **Lycopene**  **(mg 100 g^-1^ FW)** | **Flavonoids**  **(mg 100 g^-1^ FW)** | **Vitamin C**  **(mg 100 g^-1^ FW)** | **Protein**  **(U/min/mg)** |
| --- | --- | --- | --- | --- | --- | --- |
| **Control** | 59.6**^e^**  (±0.55) | 21.5**^d^**  (±0.29) | 2.16**^d^**  (±0.15) | 14.1**^e^**  (±0.15) | 15.8**^d^**  (±0.37) | 5.26**^d^**  (±0.31) |
| **Fungicide** | 87.4**^b^**  (±0.53) | 30.1**^b^**  (±0.19) | 3.56**^a-c^**  (±0.17) | 20.4**^c^**  (±0.32) | 17.4**^c^**  (±0.31) | 8.92**^ab^**  (±0.23) |
| **PP-AgNPs-50 µg/mL** | 80.1**^d^**  (±0.15) | 27.6**^c^**  (±0.23) | 3.07**^c^**  (±0.36) | 19.2**^d^**  (±0.24) | 18.4**^b^**  (±0.32) | 7.59**^c^**  (±0.29) |
| **PP-AgNPs-100 µg/mL** | 89.5**^a^**  (±0.21) | 33.5**^a^**  (±0.26) | 3.84**^a^**  (±0.26) | 21.7**^a^**  (±0.14) | 19.7**^a^**  (±0.24) | 9.54**^a^**  (±0.26) |
| **PP-AgNPs-125 µg/mL** | 87.6**^b^**  (±0.19) | 32.9**^a^**  (±0.14) | 3.75**^ab^**  (±0.27) | 21.3**^ab^**  (±0.30) | 19.5**^a^**  (±0.19) | 9.28**^ab^**  (±0.21) |
| **PP-AgNPs-150 µg/mL** | 82.3**^c^**  (±0.18) | 30.7**^b^**  (±0.17) | 3.68**^bc^**  (±0.14) | 20.9**^bc^**  (±0.28) | 19.2**^ab^**  (±0.27) | 8.46**^bc^**  (±0.36) |
| **CV (%)** | **0.74** | **1.30** | **11.6** | **2.11** | **2.73** | **5.99** |

Different letters per column indicate significant differences (P ≤ 0.05) among treatments, determined by the LSD Fisher test. Each value of data is the average of three replicates. Values with ± indicate standard error between the mean of different replicates of the same treatment. CV represents the coefficient of variation.

**Table S3.** Silver (Ag)-Content (µg/g dry weight of tissue) in the root, shoot, and fruit of tomato plants treated with PP-AgNPs (100 µg/mL), infected with *F. oxysporum* under field conditions during the years 2021 and 2022.

| **Treatment** | **Year-2021** | | | **Year-2022** | | |
| --- | --- | --- | --- | --- | --- | --- |
|  | **Root** | **Shoot** | **Fruit** | **Root** | **Shoot** | **Fruit** |
| Control (T_1_) | 3.41**^b^**  (±0.51) | 1.19**^b^**  (±0.04) | 0.014**^b^**  (±0.11) | 3.55**^b^**  (±0.55) | 1.25**^b^**  (±0.02) | 0.019**^b^**  (±0.11) |
| PP-AgNPs (T_3_) | 43.8**^a^**  (±2.74) | 29.5**^a^**  (±1.78) | 0.032**^a^**  (±0.16) | 42.1**^a^**  (±2.55) | 30.3**^a^**  (±1.52) | 0.038**^a^**  (±0.09) |

*Different letters per column indicate significant differences among treatments, determined by ANOVA and LSD-test at (P≤0.05). Values with ± indicate standard error between the mean of different replicates of the same treatment. CV represents the coefficient of variation.

**References:**

1. Ashraf, H., Anjum, T., Riaz, S. & Naseem, S. Microwave-Assisted Green Synthesis and Characterization of Silver Nanoparticles Using Melia azedarach for the Management of Fusarium Wilt in Tomato. *Front. Microbiol.* **11**, 238 (2020).

2. Cakmak, I. & Horst, W. J. Effect of aluminium on lipid peroxidation, superoxide dismutase, catalase, and peroxidase activities in root tips of soybean (Glycine max). *Physiol. Plant.* **83**, 463–468 (1991).

3. Dhindsa, R. S., Plumb-Dhindsa, P. & Thorpe, T. A. Leaf Senescence: Correlated with Increased Levels of Membrane Permeability and Lipid Peroxidation, and Decreased Levels of Superoxide Dismutase and Catalase. *J. Exp. Bot.* **32**, 93–101 (1981).

4. Cheema, S. & Sommerhalter, M. Characterization of polyphenol oxidase activity in Ataulfo mango. *Food Chem.* **171**, 382–387 (2015).

5. Sykłowska-Baranek, K. *et al.* Effect of l-phenylalanine on PAL activity and production of naphthoquinone pigments in suspension cultures of Arnebia euchroma (Royle) Johnst. *Vitr. Cell. Dev. Biol. - Plant* **48**, 555–564 (2012).

6. Livak, K. J. & Schmittgen, T. D. Analysis of Relative Gene Expression Data Using Real-Time Quantitative PCR and the 2−ΔΔCT Method. *Methods* **25**, 402–408 (2001).
